# Pan-cancer molecular signatures connecting aspartate transaminase to cancer prognosis, metabolic and immune signatures

**DOI:** 10.3389/fonc.2026.1727389

**Published:** 2026-05-20

**Authors:** Geoffrey H. Siwo, Amit G. Singal, Akbar K. Waljee

**Affiliations:** 1 Department of Learning Health Sciences, University of Michigan Medical School, Ann Arbor, MI, United States; 2 Center for Global Health Equity, University of Michigan, Ann Arbor, MI, United States; 3 Department of Pharmacology, University of Michigan Medical School, Ann Arbor, MI, United States; 4 Department of Internal Medicine, UT Southwestern Medical Center, Dallas, TX, United States

**Keywords:** AST - aspartate transaminase, cancer, GOT1, GOT2, miRNA, proteome, transcriptome

## Abstract

Serum aspartate transaminase (sAST) level is used routinely in conjunction with other clinical assays to assess liver health and disease. Increasing evidence suggests that sAST is associated with all-cause mortality and has prognostic value in several cancers, including gastrointestinal and urothelial cancers. Here, we undertake a systems approach to unravel molecular connections between AST and cancer prognosis, metabolism, and immune signatures at the transcriptomic and proteomic levels. We find that GOT1 and GOT2 expression is reversed in tumors relative to normal tissues whereby tumors residing in tissues that normally have low expression tend to have high GOT1/GOT2 expression and vice versa. Expression of GOT1 and GOT2 is associated with overall survival in several tumors across distinct tissues. At the proteomic level, expression of AST is associated with distinct pan-cancer molecular subtypes with an enrichment of specific metabolic and immune signatures. The GOT1 interactome is enriched with the targets of cancer-associated miRNAs, specifically mir34a – a promising cancer therapeutic, while the GOT2 interactome is enriched with proteins that interact with cancer-associated transcription factors. Our findings suggest that perturbations in the levels of GOT1/GOT2 within specific tissues reflect pathophysiological changes beyond tissue damage and have implications for cancer metabolism, immune infiltration, prognosis, and treatment personalization.

## Introduction

The measurement of circulating aspartate transaminase enzyme levels in serum (sAST) is commonly performed in clinical laboratories to assess liver health and damage. In humans, AST occurs as two isoenzymes encoded by distinct genes: a cytoplasmic (Glutamic Oxaloacetic Transaminase 1, GOT1) and a mitochondrial isoform (GOT2) (1). GOT1 is expressed mainly in red blood cells and the heart, while GOT2 is expressed predominantly in the liver (1). Clinical laboratory tests for AST measure its level in serum (sAST) and do not differentiate between the two isoenzymes (1).

Even though AST is typically used in the context of liver damage, the enzyme is expressed in a wide range of tissues, including in the gastrointestinal system, skeletal muscle, heart, and kidneys (2). High levels of sAST have been associated with various liver diseases including cirrhosis, metabolic dysfunction associated steatotic liver disease (MASLD) and liver hepatocellular carcinoma (LIHC) (3–5). In addition, sAST has been associated with several non-liver tumors, cardiovascular diseases, type 2 diabetes and all-cause mortality (6–8). Besides these epidemiological associations, increasing evidence from molecular studies paint a complex role of AST isoenzymes in cancer (9–13), encephalopathies (14, 15), inborn errors of metabolism (16), ischemic stroke (17) and neurodegenerative diseases such as Parkinson’s, Alzheimer’s, and Huntington’s disease (18). Epidemiologically, sAST has been associated with prognosis of cancers spanning multiple tissues including non-metastatic renal cell carcinoma (19, 20), head and neck cancer (21), gastric adenocarcinoma (22), esophageal squamous cell adenocarcinoma (23), upper urinary tract urothelial carcinoma (24), prostate cancer (25), and Pancreatic Ductal Adenocarcinoma (PDAC/PAAD) (26). While these studies have provided evidence for prognostic associations between sAST and cancer, insights into the underlying biological mechanisms are lacking.

Metabolic reprogramming, the process by which cancer cells rewire their metabolism to support the high demand for proteins, nucleotides and lipids required for rapid growth and proliferation is increasingly considered a hallmark of cancer (27). The non-essential amino acid glutamine (28) is critical for cancer cell growth and proliferation (29, 30). Glutamine is broken down into glutamate by the action of glutaminases and the resulting glutamate can be used to generate α-ketoglutarate. AST catalyzes the reversible conversion of glutamate to aspartate which provides a carbon and nitrogen source for the biosynthesis of purines, pyrimidines, amino acids and driving the malate-aspartate shuttle for redox homeostasis (31). Consequently, in some tumors, growth and proliferation are highly dependent on aspartate (32–34). GOT1 catalyzes the conversion of α-ketoglutarate and aspartate into oxaloacetate and glutamate while the GOT2 catalyzes the reverse reaction (35). GOT1 and GOT2 play critical roles in the malate-aspartate shuttle where they transfer reducing equivalents of NADH into mitochondria for oxidative phosphorylation (36). In PDAC, GOT1 has been reported as a prognostic marker (37) and functionally linked to KRAS-dependent metabolic reprogramming (9). Further, inhibition of GOT1 was found to impair pancreatic cancer growth (38, 39). GOT2 has been associated with cancer through several mechanisms (13). For example, GOT2 can traffic fatty acids leading to suppression of antitumor immunity via the peroxisome proliferator activated receptor alpha-dependent pathway (PPAR) (40, 41). In triple negative breast cancer, GOT2 overexpression was associated with increased proliferation (42–44), while in clear cell renal cell carcinoma GOT2 was downregulated and associated with poor survival and immune infiltrates (45). A gain of function mutation in GOT2 has been reported in rare neurological tumors that also harbor loss of function mutations in the malate aspartate shuttle (46, 47). Exogenous expression of GOT2 in chimeric antigen receptor (CAR)-T cells enhanced *in vitro* and *in vivo* cytotoxic activity in liver cancer (48). Collectively, these studies indicate that the associations between AST and various cancers at the epidemiological level may be driven by several molecular mechanisms.

To date, there have been no comprehensive studies involving pan-tissue transcriptomic and proteomic assessments of AST isoforms across several cancers and their association with tumor prognosis or metabolic and immune infiltration. Whether expression characteristics of GOT1/GOT2 are tissue specific and their association with tumor prognosis is mediated by metabolic and immune mechanisms remain open questions. To answer these questions, we leverage public genomic and proteomic datasets to investigate pan-cancer associations between GOT1 and GOT2 gene expression and protein levels with tumor prognosis, metabolic and immune signatures.

## Methods

### Pan-tissue, pan-cancer transcriptome analyses of AST levels

Data source for pan-tissue transcriptomes: The expression of genes encoding AST isoenzymes (GOT1 and GOT2) across tissues was based on data from the Genotype Tissue Expression portal which comprises samples from 54 non-diseased tissues of nearly 1000 individuals (GTEx Analysis Release V10 (dbGaP Accession phs000424.v10.p2) with inclusion and exclusion criteria previously described in (49). Details of GTEX V10 data generation and analysis of gene expression was previously published in the GTEX consortium paper (50).

Data pre-processing and analysis of GTEX transcriptomes: We utilized pre-processed GTEX data with RNAseq data normalized for sequencing depth and gene length resulting into log-transformed Transcripts per Million (log10 TPM + 1) (51). Data was visualized using boxplots on the GTEX portal with transcript levels of GOT1 and GOT2 represented as Log10 TPM + 1. Outliers were considered as gene expression data points that were above or below 1.5 times the interquartile range. Only bulk gene expression was considered. The expression level of each was based on a gene model in which, alternatively, spliced transcript isoforms are collapsed into single genes.

Pan-cancer transcriptomes analyses: The transcript levels of GOT1 and GOT2 in cancer cells were based on tumor and the corresponding normal tissue data from The Cancer Genome Atlas (TCGA) (52). Analyses and visualization of TCGA datasets were performed using the UALCAN resource (accessed December 3^rd^ 2023) (53, 54). The comparisons and P-value estimates of expression level differences of GOT1 and GOT2 in normal vs tumor tissue were performed using UCSC Xena platform (55).

### Pan-cancer proteomic analyses of AST levels

We leveraged previously described cancer molecular subtypes based on proteogenomic characterization of 2002 primary tumors from 14 cancers types (56) and proteomic datasets across 532 cancers spanning 6 tissue-based types (breast, colon, ovarian, renal and uterine) (57). These subtypes were obtained previously from unsupervised clustering of the proteomic data of 2002 primary tumors leading to the identification of 11 molecular subtypes (s1 to s11), each including multiple tissue-based cancers (56). In addition, we leveraged ten molecular subtypes (k1 to k10) that were previously described and found to be enriched with distinct oncogenic and metabolic pathways (57). To determine whether the tumors expressing high or low protein levels of GOT1 and GOT2 are associated with distinct tumor molecular subtypes, we used the UALCAN portal (53, 54) (accessed December 3^rd^ 2023) leveraging mass-spectrometry proteomic data across the previously described pan-cancer subtypes from the Clinical Proteomic Tumor Analysis Consortium (CPTAC) Confirmatory/Discovery cohorts (58). Statistical significance of differential expression of GOT1/2 in tumor vs. normal adjacent tissues was determined using a P-value threshold of 0.002 after Bonferroni correction for multiple hypothesis testing of an initial threshold of P < 0.05.

### Analyses of AST associations to cancer prognosis and immune infiltration signatures

#### GOT1 and GOT 2 survival analysis

Survival analysis was performed using TCGA data based on tumor gene expression levels of GOT1 and GOT2, separately from the Survival Genie portal (59). Tumors were categorized into those expressing high vs. low levels of GOT1 (GOT2) using the median gene expression value of the transcript derived from the RNA-seq normalized FPKM gene expression. The categories were then used to define tumor cohorts into high vs. low expressing groups for survival analyses using Kaplan Meir plots on Survival Genie (59). A P-value threshold of P <0.05 was used to define the significant association between GOT1 and GOT2 expression and overall survival. To adjust for the potential impact of gender and clinical stage of tumors on cancer survival outcomes associated with the expression of GOT1/2, we performed stratified analyses using Kaplan Meir Plotter (60) (KM-Plotter for pan-cancer analysis; accessed January 23^rd^ 2026).

#### Analysis of GOT1 and GOT2 associations with tumor infiltrating lymphocytes

To determine correlations between GOT1 expression (or GOT2 separately) and TILs, an implementation of the CIBERSORT algorithm (61, 62) within Survival Genie (59) was used. Briefly, for each tumor sample, the relative fraction of TIL subtypes was estimated for validated LM6 and LM22 immune cell signatures using bulk tumor FPKM gene expression data based on the CIBERSORT deconvolution method (62). The tumor’s inferred cell composition was then correlated to the expression of GOT1 or GOT2. Significant positive or negative correlation was assessed using a cutoff of P < 0.05.

#### Analysis of context-dependent effects GOT1 and GOT2 on overall survival

We hypothesized that context-dependent effects of GOT1 and GOT2 on survival outcomes in select tumors may arise from genetic dependencies with tumor mutational backgrounds. To assess genetic dependencies between GOT1 and GOT2 with TP53 and KRAS, we analyzed the co-occurrence of mutations in these genes within TCGA tumors, focusing on tumors where GOT1 and GOT2 showed differential effects on survival. Tumor mutations in these genes were obtained from TCGA using CBioportal (63, 64) (accessed January 23^rd^ 2026). Co-occurrence of mutations was computed using Log2 Odds Ratio quantifying how strongly the presence or absence of alterations in GOT1 (GOT2) are associated with the presence or absence of alterations in KRAS (TP53). Statistical significance of co-occurrence of mutations was computed using a two-sided Fisher Exact Test to obtain a P-value followed by correction for multiple hypothesis testing using Benjamini-Hochberg false discovery rate (FDR) to obtain a q-value. A q-value < 0.05 was regarded as significant.

### Analysis of AST protein-protein interaction networks

GOT1 and GOT2 protein-protein interaction networks were obtained from the BioGrid database version 4.4 (accessed March 1^st^ 2024) (65). The analysis of disease genes enriched in each network was assessed using the DisGeNET database version 7.0 (accessed August 25^th^ 2023), which contains over 1 million disease-gene associations involving 21,671 genes and 30,170 diseases, traits, and human phenotypes involving 369,554 variant-disease associations (66). The statistical significance of disease enrichments was based on a P and q-value estimate of <0.05, and only the top 10 significantly enriched diseases were considered. The enrichment of miRNA target genes in the GOT1 and GOT2 networks was determined using Enrichr (accessed August 25^th^ 2023) (67–69) and miRTarBase (accessed August 25^th^ 2023), a curated database of experimentally determined microRNA-target interactions (70, 71). A significance threshold of both P and q-value < 0.05 was applied to the miRNA enrichments, where the q-value refers to P-values corrected for false discovery rates to control for multiple hypothesis testing.

### Analysis of regulatory interactions

Two types of regulatory interactions associated with GOT1 and GOT2 protein-protein networks were assessed. The first type of regulatory interactions involved transcription factor protein-protein interactions to identify GOT1 and GOT2 interacting proteins that also interact with specific transcription factors. The protein-protein interaction networks for transcription factors were based on the literature (67–69) while GOT1 and GOT2 networks were from BioGrid version 4.4 (65). For each transcription factor, the statistical significance of enrichment of proteins in its network was estimated using a P-value and corrected for multiple hypothesis testing using Benjamini-Hochberg method to generate a q-value. Enrichments were estimated using Enrichr (67–69) and only those with P- and q-values < 0.05 were regarded as significant.

### Analysis of functional relevance of mir34a

To investigate the functional relevance of mir34a-5p identified as an miRNA whose targets are enriched in GOT1 protein-protein interaction networks, we leveraged the METAmIR34TARGET Database (72) (accessed January 22^nd^ 2026) that contains pearson correlations between transcripts levels of mir34a and other mRNAs across TCGA tumors. We then compared the distribution of Pearson correlations between transcripts levels of mir34a and all its targets vs. correlations to a subset of its targets that encode proteins interacting with GOT1. Targets of mir34a were obtained from miRTarBase 2017 (71, 73, 74)(accessed August 25^th^ 2023). A Wilcoxon rank sum test was used to determine the statistical significance of the differences in correlations between the two groups of mir34a targets for each tumor. A threshold of P < 0.05 was used followed by Bonferroni correction for multiple hypothesis testing to a P < 0.0006.

## Results

### Study overview

To investigate the association between AST (GOT1 and GOT2) in human cancers, this study undertook a pan-tissue, pan-cancer approach leveraging public genomic resources including the Genotype Tissues Expression (GTEX) resource for transcriptional datasets across normal human tissues (49, 50), The Cancer Genome Atlas (TCGA) which includes genomic and clinical datasets across 33 tumor types (75) and the Clinical Proteomic Tumor Analysis Consortium (CPTAC) resource (76, 77). Comparison of GOT1 and GOT2 expression at the transcriptomic and proteomic levels in tumors and normal samples was performed, leading to the identification of tumors where these genes are differentially expressed. To investigate the biological relevance of GOT1/2 expression with respect to cancer biology, we analyzed whether the expression of these genes differs across previously reported pan-cancer molecular subtypes characterized by proteogenomic signatures (56, 57). We performed survival analysis based on TCGA datasets to determine associations between GOT1/2 expression and overall survival of cancer patients. Finally, to investigate potential mechanisms through which GOT1/2 could impact cancer outcomes, we performed computational predictions of tumor infiltrating lymphocytes (TILs) based on tumor gene expression profiles and incorporated analyses of protein-protein interaction networks.

### Pan-tissue, pan-cancer expression of AST

**Figure 1:**
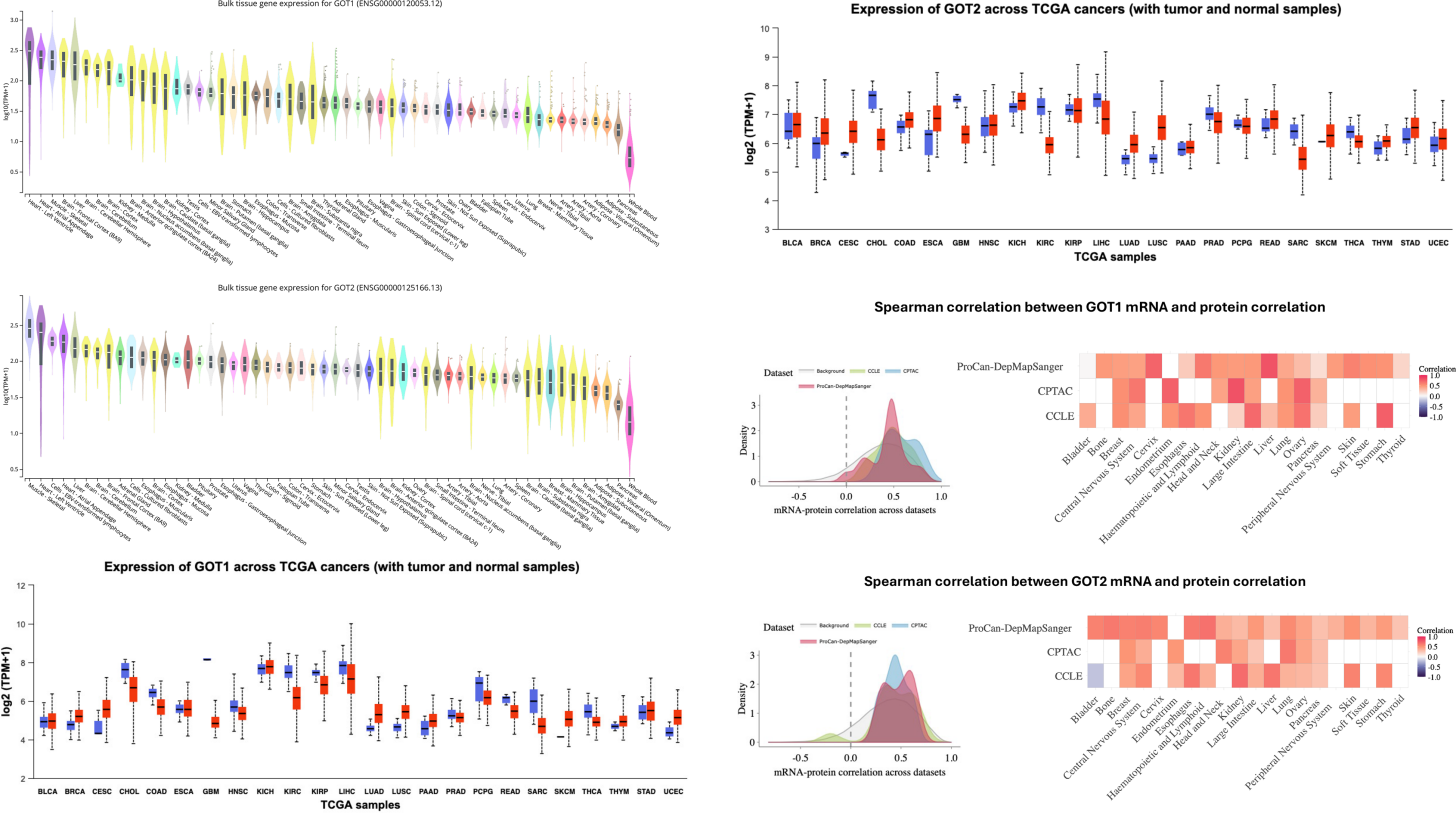
Boxplots show pan-tissue and pan-cancer transcript levels of GOT1 and GOT2 based on GTEX and TCGA datasets, respectively. Also shown are Spearman correlations between GOT1 mRNA and protein levels across different tumors based on data from ProCan-DepMapSanger, CPTAC and CCLE. Abbreviations of tumors in boxplots: BRCA, Breast cancer; BLCA, Bladder urothelial carcinoma; CESC, Cervical squamous cell carcinoma and endocervical adenocarcinoma; CHOL, Cholangiocarcinoma; COAD, Colorectal adenocarcinoma; ESCA, Esophageal cancer; GBM – Glioblastoma; HNSC, Head and Neck Squamous Cell Carcinoma; LIHC, Liver Hepatocellular carcinoma; KIRC, Kidney Renal Clear Cell Carcinoma; KIRP, Kidney Renal Papillary Cell Carcinoma; LGG, Brain Lower Grade Glioma; LIHC, Liver Hepatocellular Cancer; MASLD, Metabolic dysfunction associated steatotic liver disease; MESO, Mesothelioma; PAAD, Pancreatic Adenocarcinoma; PCPG, Pheochromocytoma and Paraganglioma; PDAC, Pancreatic Ductal Adenocarcinoma; PRAD, Prostate Adenocarcinoma; LAML, Acute Myeloid Leukemia; LUAD, Lung adenocarcinoma; LUSC, Lung Squamous Cell Carcinoma; UCEC, Uterine Corpus Endometrial Carcinoma; READ, Rectal Adenocarcinoma; SARC, Sarcoma; SKCM, Skin Cutaneous Melanoma; THCA -Thyroid Carcinoma; THYM, Thymoma; UCEC, Uterine Corpus Endometrial Carcinoma.

To assess the expression level of AST across multiple tissues, we queried the GTEX resource. GOT1 and GOT2 expression was evident in multiple tissues with the highest levels of expression in skeletal muscle, the heart, liver and specific regions of the brain (**Figure 1**). The lowest expression level was in whole blood. Tissues expressing high levels of GOT1 also tended to express high levels of GOT2.

Given the extensive pan-tissue expression of GOT1 and GOT2, we performed an analysis of their expression across multiple cancers in TCGA. Relative to the normal adjacent tissue, GOT1 expression was higher in breast cancer (BRCA, P < 0.001), cervical squamous cell carcinoma and endocervical adenocarcinoma (CESC, P = 0.12), lung adenocarcinoma (LUAD, P = 1.351e-13), Lung Squamous Cell Carcinoma (LUSC, P = 1.733e-24), Thymoma (THYM, P = 0.1559) and Uterine Corpus Endometrial Carcinoma (UCEC, P = 1.714e-12). However, GOT1 was expressed at lower levels in Cholangiocarcinoma (CHOL, P = 8.212e-11), Colorectal Adenocarcinoma (COAD, P = 1.998e-15), glioblastoma (GBM, P < 0.001), Head and Neck Squamous Cell Carcinoma (HNSC, P < 0.001), Kidney Renal Clear Cell Carcinoma (KIRC, P < 0.001), Kidney Renal Papillary Cell Carcinoma (KIRP, P = 0.0004776), Hepatocellular Carcinoma (LIHC, P = 4.127e-12), Pheochromocytoma and Paraganglioma (PCPG, P = 0.1990), Rectal Adenocarcinoma (READ, P = 4.797e-8), sarcoma (SARC, P = 0.003331) and Thyroid Carcinoma (THCA, P = 0.02) (**Figure 1**). After Bonferroni correction (P < 0.002), GOT1 expression in tumors vs. normal adjacent tissue was not significant for PAAD, PCPG, THYM, CESC, SARC and THCA.

Relative to the adjacent normal tissue, GOT2 expression was highly expressed in BRCA (P << 0.001), CESC (P = 0.1031), LUAD (P = 1.933e-13), LUSC (P = 4.948e-62), Pancreatic Adenocarcinoma (PAAD, P = 0.5721) and UCEC (P = 0.03423) but expressed at reduced levels in CHOL (P = 2.349e-17), GBM (P = 1.601e-9), KIRC (P << 0.001), LIHC (P = 1.110e-16), Prostate Adenocarcinoma (PRAD, P = 0.003360) and SARC (P = 0.005591). For both GOT1 and GOT2, tumors whose resident tissues normally have high expression of these genes tended to have low expression and vice versa. For example, while the liver and muscle are among the top tissues expressing high levels of GOT1 and GOT2 when compared to all other tissues in GTEX, tumors from these tissues had downregulated levels of expression of these genes relative to the corresponding normal tissue. Conversely, in lungs and uterus - tissues with low constitutive expression of GOT1 and GOT2-, these genes were upregulated in the tumor compared to the corresponding normal tissue. After Bonferroni correction for multiple hypothesis testing (P < 0.002), GOT2 expression in tumors vs. normal adjacent tissue was not significant for CESC, PAAD, UCEC, PRAD and SARC.

**Figure 2:**
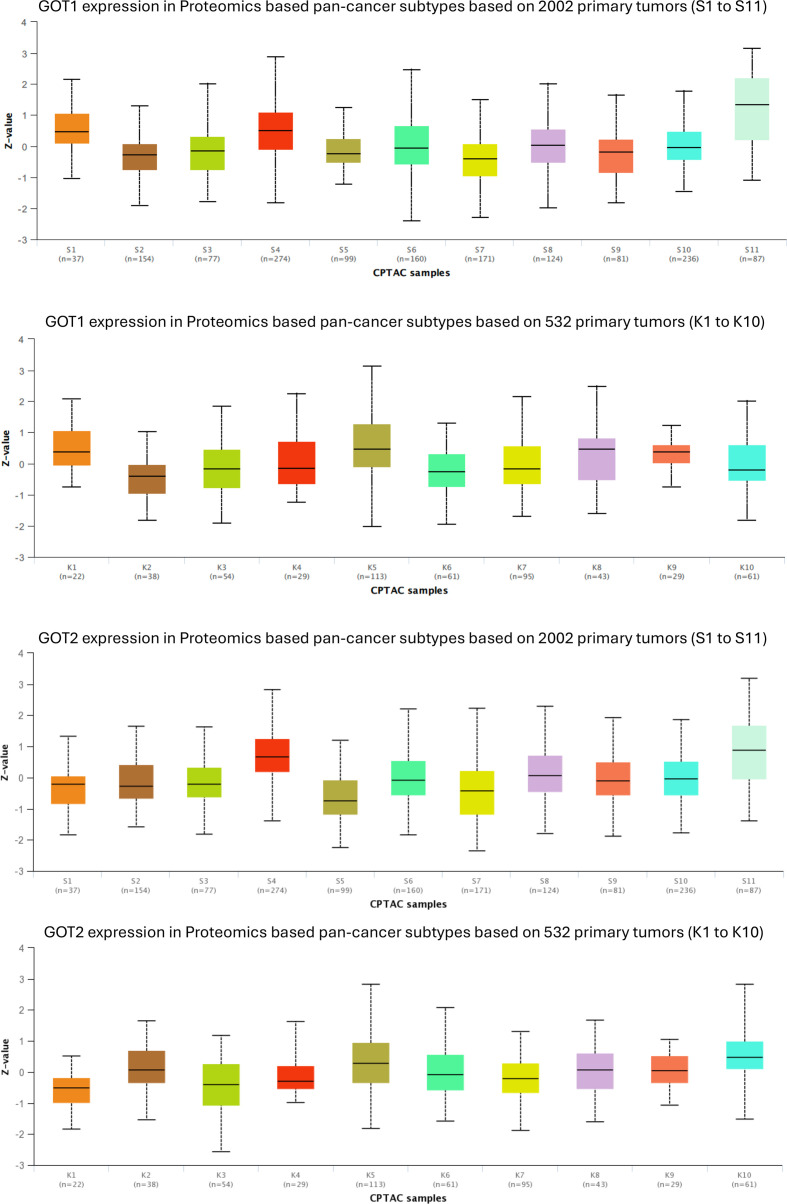
GOT1 and GOT2 expression in previously defined pan-cancer molecular subtypes based on proteogenomic characterization. See **Supplementary Tables 1** to **4** for P-values. K1 to K10 and S1 to S11 are previously described pan-cancer subtypes based proteogenomic signatures of 532 tumors (57) and additional set of 2002 tumors (56). Figures produced using UALCAN resource (53, 54).

To assess whether GOT1 and GOT2 transcript levels are predictive of protein levels of the isoenzymes across tumors, we leveraged data from OnCorr (78), a large resource of mRNA-protein correlations based on datasets from ProCan-DepMapSanger (79), CCLE (80) and CPTAC (76, 77) (**Figure 2**, lower panels). The mean Spearman Correlation coefficient, *r*, between GOT1 mRNA and protein levels is positive across all tumors in each of the cancer datasets (mean *r* in ProCan-DepmapSanger = 0.43; mean *r* in CCLE = 0.52; mean *r* in CPTAC = 0.26). The lowest Spearman Correlation between GOT1 mRNA and protein levels was observed for ProCan-DepMapSanger bladder cancer samples (mean *r* = 0) and ProCanDepMapSanger pancreatic cancer samples (mean *r* = 0.14). Similarly, GOT2 mRNA and protein levels were generally positively correlated (mean *r* in ProCan-DepMapSanger = 0.48; mean *r* in CCLE = 0.46; mean *r* in CPTAC = 0.26). The only tumor in which GOT2 mRNA and protein levels were negatively correlated was in bladder cancer samples from CCLE (mean *r* = -0.2) but bladder cancer samples in ProCan-DepMapSanger exhibited a strong positive correlation (mean *r* = 0.59). Thus, GOT1 and GOT2 mRNA and protein levels are significantly positively correlated implying that GOT1 and GOT2 mRNA levels can be used as proxies for their protein levels.

### AST protein levels in pan-cancer proteomic subtypes

Given the extensive differential expression of GOT1 and GOT2 across multiple tumors coupled with prior studies linking the expression of these genes to tumor metabolic rewiring (9, 12, 39, 44, 81, 82), we next investigated the association between GOT1 and GOT2 protein levels in a set of 10 (K1 to K10) and 11 (S1 to S11) previously described pan-cancer molecular subtypes across a set of 532 tumors (57) and additional set of 2002 tumors (56) based on proteogenomic signatures.

The top pan-cancer subtypes associated with high expression of GOT1 were K5, K8 and K1, and S11 and S4 (**Figure 2**). K5 subtypes were previously associated with tricarboxylic acid cycle (TCA) and oxidative phosphorylation as well as with targets of MYC, YAP1, and Ras pathway (57). K8 was associated with the Ras pathway, while K1 with the pentose phosphate pathway and glycolysis (57). S4 and S11 proteomic signatures were previously associated with increased activation of fatty acid metabolism, glycolysis and gluconeogenesis, pentose phosphate, TCA cycle and oxidative phosphorylation (56), consistent with the biochemical role of AST.

In contrast, the cancer subtypes with the least expression of GOT1 were K2 and K6, and S2 and S7. Based on previous studies (57), K2 subtypes were associated with MYC targets, K6 with hypoxia, WNT and Epithelial-to-Mesenchymal Transition (EMT). S2 were associated with gene signatures indicating the presence of T-cells and a higher expression of immune checkpoint pathway genes and S7 associated with “axon guidance” and “frizzled binding genes” (56). Pairwise comparisons of GOT1 expression in the different pan-cancer subtypes revealed that GOT1 is especially differentially expressed in S7 vs. S11 (P = 3.2e-22), S4 vs. S7 (P = 1.1e-19) and S2 vs. S11 (P = 1.3e-19). **Supplementary Tables 1** and **2** provides P-values comparing GOT1 expression in all the pairs of pan-cancer “S” (**Supplementary Table 1**) and “K” (**Supplementary Table 2**) sub-types.

The top pan-cancer subtypes associated with high expression of GOT2 were S4 and S11 subtypes (also associated with GOT1), and K10 and K5 (also associated with GOT1) subtypes (**Figure 2**). The K10 pan-cancer subtype was previously characterized by elevated ER-related proteins and steroid biosynthesis pathway protein, while the K5 subtype was associated with YAP1 and MYC targets, and Ras pathway (57).

The pan-cancer subtypes S5 and K3 had the lowest expression of GOT2 transcript levels (**Figure 2**). S5 subtype tumors have been associated with B cells, mast cells, eosinophils and elevated expression of complement pathway genes (56). In contrast, the K3 subtype was previously associated with hypoxia and EMT signatures. Furthermore, K3 was also enriched with gene signatures for markers of mast cells, macrophages, eosinophils, neutrophils and the complement system (57).

Pairwise comparisons of GOT2 expression in the different pan-cancer subtypes revealed that GOT2 is especially differentially expressed in S4 vs. S5 (P = 9.7e-32), S4 vs. S7 (P = 1.8e-28) and S4 vs. S10 (P = 9.3e-22). **Supplementary Tables 3** and **4** provides P-values comparing GOT2 expression in all the pairs of pan-cancer “S” (**Supplementary Table 3**) and “K” (**Supplementary Table 4**) sub-types.

### AST transcript levels, tumor prognosis and immune infiltration signatures

**Figure 3:**
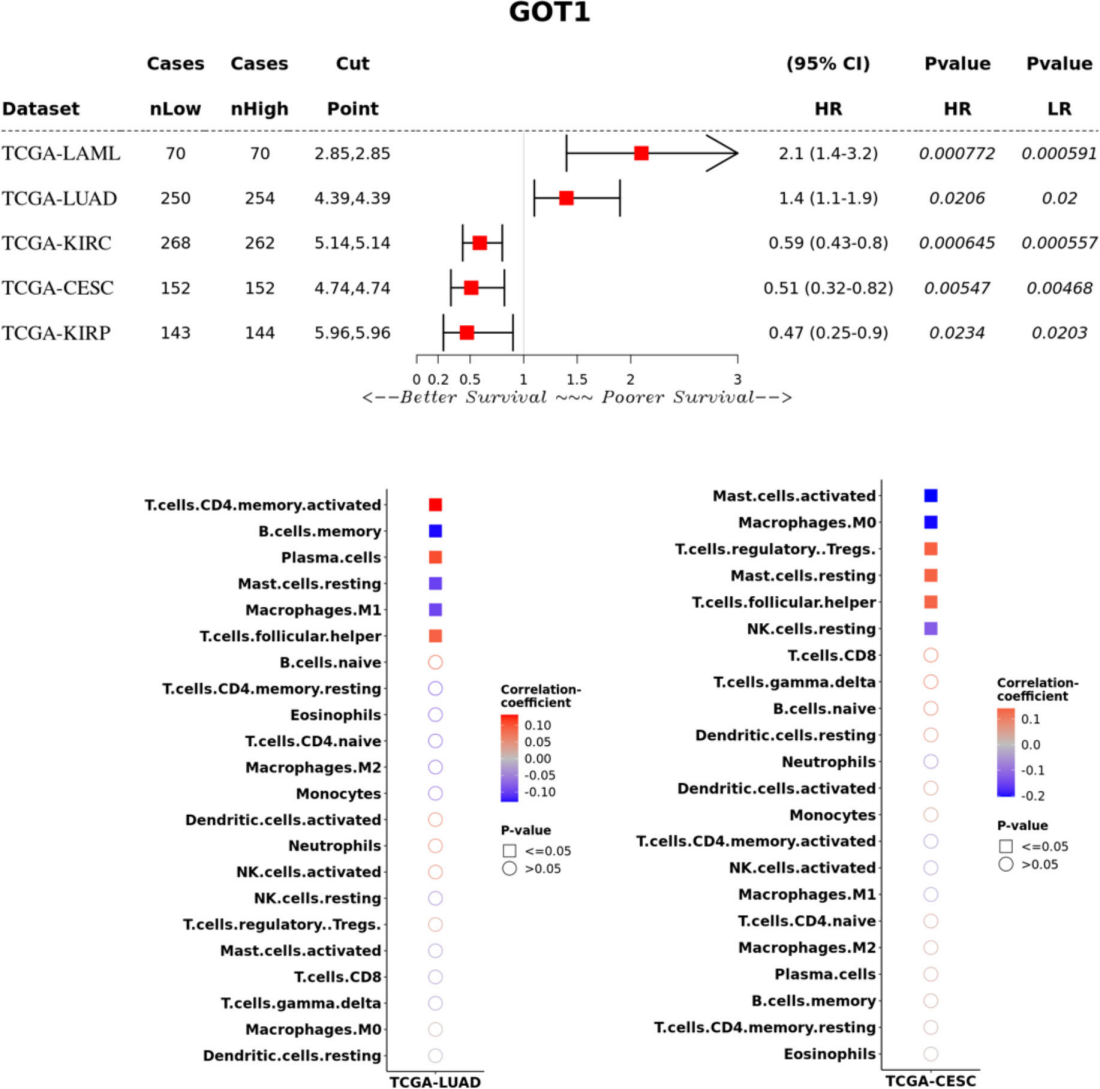
GOT1 expression and associations to overall survival and immune cell infiltration. For the immune cell infiltration analyses (lower panel), the color scale represents the correlation between GOT1 expression and enrichment of different TILs in LUAD and CESC based on levels of immune cell infiltrates inferred by CIBERSORT. Figures produced using Survival Genie (59).

We examined the association of GOT1 and GOT2 transcript levels with prognosis in TCGA tumors using patient groups classified as expressing high or low levels of either gene. High expression of GOT1 was significantly associated with poorer survival in LUAD (hazard ratio, HR = 1.4; confidence interval, CI = 1.1-1.9; P = 0.02) and LAML (HR = 2.1; CI = 1.4-3.2; P = 0.0008), and better survival in CESC (HR = 0.51; CI = 0.32-0.82; P = 0.005), KIRC (HR = 0.59; CI = 0.43-0.8; P = 0.0006) and KIRP (HR = 0.47; CI = 0.25-0.9; P = 0.02) (**Figure 3**). However, GOT1 expression was not associated with survival in several cancers where these genes were differentially expressed relative to normal tissue.

To adjust for gender and clinical stage of tumors, we performed stratified analyses of the association between GOT1 and overall survival. In KIRC, high GOT1 expression was associated with better survival in males (HR = 0.5, CI = 0.34-0.74, P = 0.0039; **Supplementary Figure 2**) but not in females (P = 0.05). In the case of clinical stage of tumors, GOT1 expression was not associated with survival in all the clinical stages (Grade 2, 3 and 4) for which sufficient data were available. In LUAD, there was no significant association between GOT1 and survival when stratified by either sex (**Supplementary Figure 2**). In the case of KIRP, high expression of GOT1 was associated with better survival in females (HR = 0.16, CI = 0.03-0.71, P = 0.006) but not males (P = 0.43; **Supplementary Figure 2**). For CESC, sufficient data was only available for Grade 2 and 3 tumor stages. No significant association was obtained between GOT1 expression and CESC in either Grade 2 or 3 tumors. Due to insufficient data for tumor grade in LUAD and KIRP, we were unable to perform stratified analyses to investigate the association of GOT1 and tumor grade with survival in these tumors. Although previous studies found a prognostic association between GOT1 and prognosis in PAAD (37), we did not find this association to be significant (P = 0.43) but PAAD patients with high levels of GOT1 transcripts tended to have longer survival (**Supplementary Figure 1**). The lack of statistical significance could be due to the small sample size of PAAD tumors in TCGA (n = 178) and the Kras-dependent effects of GOT1 (9) may confound the analysis.

To investigate the potential role of tumor infiltrating lymphocytes (TILs) in the distinct association between GOT1 and survival in LUAD and CESC, we examined the correlation between GOT1 expression and TILs based on gene expression signatures. In both tumors, GOT1 expression was positively correlated with follicular helper T-cells. In LUAD, GOT1 was positively correlated with activated CD4+ memory T-cells and plasma cells, and negatively correlated with memory B-cells, resting mast cells and M1 macrophages (**Figure 3**). In contrast, GOT1 expression in CESC was positively correlated to regulatory T-cells (Tregs) and resting mast cells, and negatively correlated to activated mast cells, M0 macrophages and Natural Killer (NK) cells (**Figure 3**). The positive correlation between GOT1 and Tregs in CESC is consistent with a recent report demonstrating that GOT1 expression promotes the differentiation of Tregs (83, 84). While Tregs are generally associated with poorer survival outcomes due to their immunosuppressive nature (85), high GOT1 expression in CESC was associated with better overall survival (**Figure 3**). These results suggest that GOT1 may have context-specific effects on tumor immune responses. We provide some potential hypotheses for this counterintuitive results in the discussion section.

Another potential factor contributing to the differential association of GOT1 to survival in LUAD vs. CESC could be due to distinct mutational backgrounds of these tumors. To test, this hypothesis, we considered potential genetic dependencies between GOT1 and frequently mutated genes in tumors – KRAS and TP53- based on TCGA PanCancer Atlas data on CBioportal (63, 64). In LUAD, we found that GOT1 mutations while rare (n= 23, ~5% of LUAD cases) tend to co-occur with KRAS mutations but not TP53 mutations. Specifically, 14 out of 23 (60.9%) LUAD patients with GOT1 mutations also had KRAS mutations (log2 odd ratio = 1.93, P = 0.003, q = 0.014). TP53-GOT1 mutation co-occurrence was not significant after correction for multiple testing (log2 odds ratio = -1.23, P = 0.034, q = 0.14). Similarly, in CESC patients (only females), we did not find significant co-occurrence of GOT1 and KRAS mutations (log2 odds ratio = 2.79, P = 0.05, q = 0.1) or GOT1 and TP53 mutations (log2 odds ratio = -3, P = 1, q = 1). However, these results are limited given that KRAS mutations were rare in the cohort of CESC patients (n= 16 out of 278 or 6%). None of the 16 CESC patients with KRAS mutations also had a GOT1 mutation (n= 5 for samples with GOT1 mutation or 2%).

**Figure 4:**
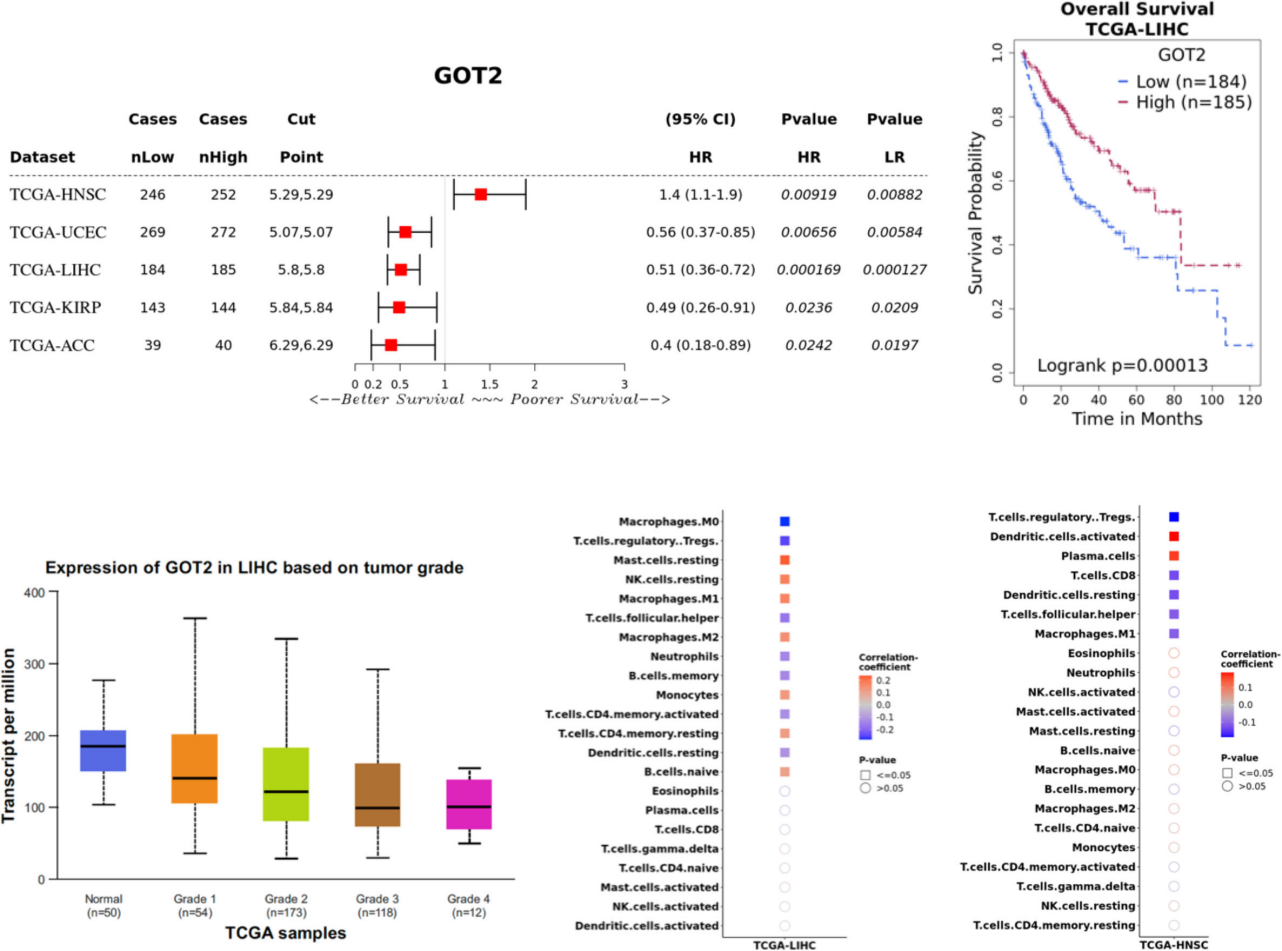
GOT2 expression and associations to overall survival and immune cell infiltration. Sample sizes for the survival curve analysis are embedded in the survival curve plot (upper right panel, n = 184 for patients with low GOT2 expression; n = 185 for patients with high GOT2 expression). For the immune cell infiltration analyses (lower right panel), the color scale represents the correlation Figures produced using Survival Genie (59) and UALCAN (53,54). between GOT2 expression and enrichment of different TILs in LIHC and HNSC based on levels of immune cell infiltrates inferred by CIBERSORT.

Next, we investigated the association between GOT2 expression and overall survival. High expression level of GOT2 was associated with better overall survival in LIHCs (HR = 0.51; CI = 0.36-0.72; P = 0.0002), adrenocortical carcinoma/ACC (HR = 0.4; CI = 0.18-0.89; P = 0.02), KIRP (HR = 0.49; CI = 0.26-0.91; P = 0.02) and UCEC (HR = 0.56; CI = 0.37-0.85; P = 0.007), and poorer survival in head and neck cancer (HNSC) (HR = 1.4; CI = 1.1-1.9; P = 0.009), mesothelioma (MESO) (HR = 2.3; CI = 1.4-3.7; P = 0.001), Brain Lower Grade Glioma (LGG) (HR = 1.6; CI = 1.1-2.3; P = 0.012), and Acute Myeloid Leukemia (LAML) (HR = 1.6; CI = 1-2.4; P = 0.04; **Figure 4**; **Supplementary Figure 1**). GOT2 expression was not associated with survival in several cancers where these genes were differentially expressed relative to normal tissue.

To adjust for gender and clinical stage of tumors, we performed stratified analyses of the association between GOT2 and overall survival. In LIHC, GOT2 expression was not associated with survival in females (P = 0.49) but high expression of GOT2 in males was associated with better survival (HR = 0.37, CI = 0.23-0.6), P = 2.1e-05). GOT2 expression had no association to survival in Grade 1 or 2 (P = 0.08) or 3 tumors (P = 0.13, **Supplementary Figure 2**) in LIHC. In the case of KIRP, high GOT2 expression was associated with better survival in males (HR = 0.44, CI = 0.21-0.94, P = 0.028) but not females (P = 0.14, **Supplementary Figure 2**). For HNSC, GOT2 expression was not associated with survival in females (P = 0.07) or males (P = 0.05). Similarly, in UCEC (only female patients), GOT2 expression was not associated with survival in Grade 1 (P = 0.13) or 2 tumors (P = 0.47). However, high GOT2 expression was associated with better survival in Grade 3 tumors (HR = 0.51, CI = 0.31-0.83, P = 0.006, **Supplementary Figure 2**).

The prognostic associations between GOT2 and LIHC are in alignment with genetic studies in which GOT2 knockdown was reported to enhance the proliferation, migration, and invasion of LIHC cells *in vitro* and in mouse models (82, 86). Furthermore, CAR-T cells engineered to express GOT2 had increased metabolism and anti-tumor activity (48). Analysis of inferred TILs in LIHC revealed positive correlation between GOT2 and mast cells, NK cells, M1 and M2 macrophages, monocytes, CD4+ resting T-cells and naïve B-cells. In contrast, GOT2 was negatively correlated with M0 macrophages, Tregs, follicular T-helper, neutrophils, memory B-cells, activated CD4+ T-cells and resting dendritic cells (**Figure 4**). Since HNSCs are the only tumors in which a high GOT2 expression was associated with poor overall survival, we assessed the correlation between GOT2 expression and TILs. HNSC tumors had distinct patterns of TILS compared to LIHC and exhibited a positive correlation between GOT2 expression and activated dendritic cells and plasma cells (**Figure 4**).

Next, we investigated whether genetic dependencies between GOT2 with TP53 or KRAS may underly the distinct associations it has with survival in HNSC vs. UCEC, LIHC, KIRP and ACC. GOT2 had significant co-occurrence with KRAS mutations in UCEC (Log2 OR = 1.78, P = 0.013, q = 0.02). 12 out of 18 (67%) UCEC patients with GOT2 mutations also had a mutation in TP53 which was present in 193 out 509 (38%) UCEC patients. In contrast, only 2 out 18 (11%) patients with GOT2 mutations had a KRAS mutation (n = 103 patients, Log2 OR = -1.05, P = 0.55, q = 0.55). GOT2 mutations were extremely rare in LIHC (n = 1 out of 360 patients), KIRP (n = 2 out of 660 patients) and ACC (n = 4 out of 92 samples) thereby limiting the analysis of potential genetic dependencies between GOT2 and TP53/KRAS in these tumors. These results present preliminary findings and future studies with larger sample sizes are needed to establish the significance of these findings.

### AST protein-protein networks reveal physical interactions with tumor oncogenes and suppressors

**Figure 5:**
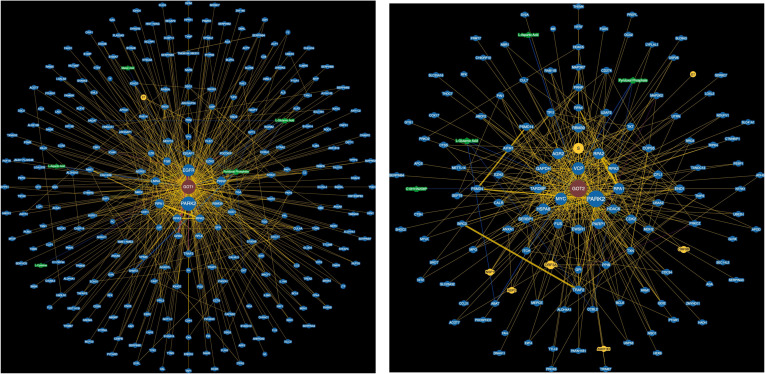
GOT1 and GOT2 protein-protein interaction networks from BioGrid.

We hypothesized that potential mechanisms underlying GOT1 and GOT2 associations could arise from protein-protein interactions involving other proteins that participate in cancer related molecular pathways. Therefore, we examined public protein-protein interaction data from the BioGrid database for GOT1 and GOT2 interactomes (**Supplementary Tables 5**, **6**; **Figure 5**). The GOT1 subnetwork from BioGrid contains 269 nodes, of which 263 correspond to distinct proteins and five correspond to small molecules (**Supplementary Table 5**; **Figure 5**). In contrast, the GOT2 subnetwork contains 90 proteins and four small molecules (**Supplementary Table 6**; **Figure 5**). Interestingly, the top hub in both subnetworks is Parkin, a ubiquitin ligase encoded by the PARK2 gene in which mutations were first found to cause autosomal recessive juvenile parkinsonism (87, 88). In addition to its role in the survival of neurons (15), PARK2 is a tumor suppressor (89) and has been associated with several types of cancer, including glioblastomas (90), lung (91), breast (92), ovarian (91), colorectal (93) and liver cancers (94).

In the GOT1 subnetwork, the presence of EGFR is notable as it is the second most connected gene in this subnetwork (**Figure 5**). EGFR is a well-studied tumor oncogene mutated and/or expressed at high levels in several cancers, especially cancers of the head and neck (95), lung (96,) and the gastrointestinal system (97–99). In addition, EGFR is the target of several approved cancer therapies and contributes to cancer drug resistance (98, 100, 101). The GOT1 network also contains GRB2, which binds to EGFR and stimulates KRAS signaling (102, 103). EGFR and GRB2 are also entry co-factors for the hepatitis B (HBV) (104) and C viruses (HCV) (105) which contribute to liver cancer (106, 107). The GOT2 subnetwork includes other critical cancer associated genes: MYC- an important transcription factor overexpressed in many tumors, and PARP1, an enzyme involved in the error-prone DNA repair pathway microhomology-mediated end joining (MMEJ) and a therapeutic target (108, 109). The protein-protein interactions between both GOT1 and GOT2 with several cancer associated genes provide a potential mechanism through which these genes may be associated with cancer.

To perform an unbiased assessment of potential disease associations between proteins in the GOT1 and GOT2 networks, we analyzed disease enrichments using gene-disease associations from the Disease Gene Network database (DisGeNET) (66). GOT1 interaction partners are highly enriched with proteins associated with dermatologic disorders including atopic dermatitis, contact hypersensitivity and acanthosis, and several cancers including adenocarcinoma of the lung and squamous cell carcinoma of the esophagus (**Supplementary Table 7**). In contrast, GOT2 partners are enriched with cancers of the liver, lung and brain (**Supplementary Table 8**). We provide detailed list of GOT1 and GOT2 interaction partners that are involved in the enriched diseases in **Supplementary Tables 7** and **8**.

### AST protein-protein networks are enriched with interaction targets of cancer associated transcription factors and miRNAs

We hypothesized that associations between GOT1 and GOT2 with cancer related biological processes may be mediated through shared protein-protein interactions with transcription factors. Therefore, we assessed whether the protein-protein interactomes of specific transcription factors are enriched with proteins in the GOT1 and GOT2 networks (**Supplementary Tables 9**, **10**). For both GOT1 and GOT2 networks, Estrogen Receptor 1 (ESR1) and Activating Transcription Factor 2 (ATF2)- a driver of tumor aggressiveness (110) were ranked in the top 3 transcription factors whose partners were enriched (P < 0.05 in all cases; **Supplementary Tables 7**, **8**). While the transcription factor USF2 (Upstream Stimulatory Factor 2, also associated with several cancers (111)) was ranked second for GOT1, the overlap between its network and that of GOT2 was ranked much lower (rank = 21; P = 0.002). Notably, the tumor suppressor TP53 was ranked second for the GOT2 network (P = 1.6e-09) but no significant enrichment of its partner proteins was found for GOT1 at P < 0.05. Surprisingly, some of the metabolic enzymes in the GOT2 protein-protein interaction network also interact with P53. Specifically, GAPDH, NQO1, COX17, TX interact with both TP53 and GOT2 (**Supplementary Table 8**). These interactions could impact cancer progression through several ways. For example, mutant P53 has been shown to prevent the nuclear translocation of GAPDH thereby enhancing glycolysis in cancer cells and inhibiting cell death mechanisms mediated by nuclear GAPDH (112, 113), NQO1 stabilizes P53 (114, 115) and TX enhances the binding of P53 to DNA (116).

Next, we assessed the GOT1 and GOT2 networks for targets of miRNAs and identified several miRNAs whose targets are enriched in these networks (**Supplementary Tables 11**, **12**). For GOT1, the targets of mir-34a-5p (P = 1.4e-08) and mir-124-3p (P = 4.7e-07) were enriched while for GOT2, mir-4476 (P = 0.00005) and mir-6876-5p (P = 0.00005) targets were enriched. mir-34a-5p is a tumor suppressor and has been associated with several cancers (117, 118) and diseases such as psoriasis (119), MASLD (120), MASH (121), obesity (122), insulin resistance (123, 124) and osteoarthritis (125). This miRNA is a transcriptional target of P53 and regulates P53 directly by targeting TP53 mRNA (126) as well as indirectly by targeting the mRNAs encoding negative regulators of P53 including HDM4 -a potent negative regulator of P53, resulting in a positive feedback loop (127). mir-124-3p is associated with several cancers including LIHC, breast and gastric cancers (128) as well as neurodegenerative diseases such as Parkinson’s and Alzheimer’s disease (129). mir-4476 enriched with GOT2 network members has been associated with gliomas (130) and the biological relevance of mir-6876-5p is currently unknown. These results suggest that GOT1 and GOT2 are functionally associated with biological processes in cancer through multiple mechanisms.

### Associations between mir34a transcript levels and the GOT1 interactome varies across tumors

To further assess the potential role of mir34a-5p on GOT1 protein-protein interaction networks as an additional mechanism of its previously reported tumor suppressor activity, we compared the correlation between mir34a and all its targets (based on miRTarBase 2017 (71, 73, 74)) for each cancer type in TCGA vs. its correlation to the subset of its targets encoding proteins in the GOT1 protein subnetwork. Interestingly, for a small set of cancer types, the distribution of correlations between mir34a and its subset of targets represented in the GOT1 subnetwork tended to be negative relative to the correlation to all its targets (Bonferroni corrected P-value < 0.002, **Supplementary Figure 3**). Tumors showing more negative correlation were PAAD (P = 0.0004), LUSC (P = 4.716e-06), LGG (P = 0.001), HNSC (P = 0.0002), CESC (P = 0.0004), UCEC (P = 3.94e-06). These results suggest that in these cancers, mir34a selectively modulates GOT1 protein subnetworks, potentially contributing to the associations between GOT1 and tumor metabolism in these cancers.

## Discussion

### Pan-cancer expression characteristics and tissue specificity of GOT1/GOT2

In this study, we performed a pan-tissue and pan-cancer analysis of GOT1 and GOT2 transcript and protein levels using publicly available transcriptome and proteomic datasets. GOT1 and GOT2 transcripts were detectable in several tissues though the expression of these genes was particularly high in the skeletal muscle, heart, liver and brain (**Figure 1**). Overall, tumors located in tissues that express low levels of GOT1 and GOT2 under normal conditions were characterized by a higher expression of these genes and vice versa. Thus, it appears that relative to their normal corresponding tissue, tumors have a reversed expression of GOT1 and GOT2.

To investigate the potential biological significance of GOT1 and GOT2 expression in tumor biology, we assessed the expression of these genes in previously described pan-cancer molecular subtypes defined using proteomic data (56, 57). At the regulatory level, our results show that high proteomic levels of GOT1 as well as GOT2 are associated with gene expression signatures of MYC, YAP1 and K-Ras, important players in several cancers. GOT1 and GOT2 tumors are also associated with metabolically similar pan-cancer subtypes characterized by gene expression signatures of the TCA cycle and oxidative phosphorylation. MYC, YAP1 and K-Ras are known to regulate several metabolic processes and have been linked to metabolic reprogramming in many tumors (131, 132). Thus, the core regulatory programs identified in this study as associated with tumors with high levels of proteomic GOT1 and GOT2 may underlie the enriched metabolic pathway signatures.

### Mechanistic associations between GOT1/GOT2 and tumor metabolism and immunity

Our results suggest that GOT1 and GOT2 expression are associated with distinct immune infiltration patterns that are tissue dependent. Low GOT1 expression was associated with gene expression signatures indicating the presence of T-cells and the expression of immune checkpoint genes, while low GOT2 expression was associated with signatures of various immune cells including mast cells and eosinophils. Among TCGA tumors, high GOT1 expression was associated with poor overall survival in LUAD and LAML and better prognosis in CESC, KIRP and KIRC. In contrast, high GOT2 expression was associated with better prognosis in liver cancer (LIHC), UCEC, KIRP and ACC, and poor prognosis in MESO, HNSC, LGG and LAML. A recent study found that GOT1 promotes the differentiation of Tregs (83, 84) which provides a possible mechanistic link through which GOT1 may influence prognosis. Although a high GOT1 expression in CESC was associated with increased Treg infiltration (**Figure 3**), which generally diminish antitumor immunity, multiple competing factors that interact to influence prognosis could counter this. For example, within CESC tumors, the negative effects of Tregs could be countered by a higher level of T follicular helper cells and resting mast cells, alongside lower levels of activated mast cells and M0 Macrophages, which collectively create a more immunocompetent environment. T-follicular help cells have been associated with favorable outcome in solid organ tumors of non-lymphocytic origin (133) including cervical cancer (134), activated mast cells were associated with poor outcomes in CESC (135) and M0 macrophages have been associated with poor outcomes in several cancers including CESC (136).

Our results show that in the case of liver cancer (TCGA LIHC), a high GOT2 expression was associated with an overall immunocompetent tumor environment as several classes of immune cells enriched in high GOT2 expressing tumors have been associated with better prognosis. Specifically, in our results, high GOT2 expression was positively associated with increased infiltration by immune cells that were previously associated with better prognosis including resting mast cells, resting NK cells, M1 macrophages, resting memory CD4 T-cells (137) and naïve B-cells (138) as well as those that have been associated with poor prognosis in liver cancer such as M2 macrophages (139) and monocytes (140). A high GOT2 expression was negatively correlated to immune cells associated with poor prognosis including Tregs and M0 macrophages (141), as well as to those previously associated with better prognosis – CD4+ memory T-cells (142), and dendritic cells (143). Thus, even though high GOT2 expression was associated with some lymphocyte subsets that generally have a negative impact on prognosis, the overall prognosis of LIHC patients with high GOT2 expression may have a better outcome. The association between GOT2 with immune infiltrating cells could have implications in the pathology of other diseases accompanied with dysfunctional expression of GOT2 such as MASH and MASLD. Functional validation of GOT1/GOT2 impact on immune infiltration will be critical in establishing the biological significance of these observations, for example through knockdown experiments.

### Mechanistic roles of GOT1/GOT2 protein-protein interactions in tumor biology

To gain deeper biological insights into potential connections between the biological functions of GOT1 and GOT2, we leveraged experimentally validated and manually curated human protein-protein interactions from the BioGrid database. The direct interaction between GOT1 and GOT2 with known tumor suppressors and oncogenes including EGFR and MYC is notable but further experimental validation of these interactions is needed as many protein-protein interaction data are obtained from noisy, high-throughput methods which presents a limitation of our study. The EGFR-GOT1 interaction in BioGrid is based on the results of a study that performed targeted characterization of EGFR interaction using a mammalian yeast-two-hybrid system, identifying a set of 87 EGFR interacting proteins (144) while the MYC-GOT2 interaction was obtained from SILAC immunoprecipitation of the targets of the ubiquitin dependent ATPase Valosin-Containing Protein (VCP) which degrades several proteins including c-Myc, via the ubiquitin-proteasome pathway (145). Independent of the protein-protein interaction networks, our analyses found that pan-cancer molecular subtypes expressing high GOT1 and GOT2 were associated with both Ras and MYC gene expression signatures, providing an orthogonal source of evidence for functional associations between GOT1 and GOT2 with Ras (a component of the EGFR signaling cascade) and Myc. The protein-protein interaction data also show that GRB2, an adaptor protein that relays EGFR signals to Ras, interacts with GOT1. Interestingly, the GOT1-GRB2 interaction is among protein-protein interactions that were reported as rewired in colorectal cancer cells expressing the mutant KRAS^G13D^ in which the interaction was only present in cells expressing high levels of mutant KRAS (146).

Our analysis of shared protein-protein interactions between transcription factors and GOT1 and 2 revealed key transcription factors including ESR1, TP53, ATF2 and USF2 that have all been associated with various cancers. ESR1 is frequently mutated in estrogen receptor (ER) positive and ER-resistant breast cancers which show increased dependence on glutamine (147). Furthermore, GOT1 and GOT2 were found to be critical for the growth of wild-type TP53 expressing cells under glutamine starvation (148). ATF2 is a transcription factor that is phosphorylated during starvation of essential amino acids, resulting into increased transcription of amino acid-regulated genes (149). Although glutamine is nonessential for normal cells, cancer cells are addicted to glutamine (150) and P53 is a critical regulator of adaptation of cancer cells to glutamine deprivation (151), a situation created in tumors by the increased utilization of this amino acid. Thus, the protein-protein interaction networks reinforce the role of GOT1 and GOT2 in glutamine adaptation in cancer.

### Potential clinical translation value

Our results show that GOT1 and GOT2 have potential prognostic value in specific tumors including LIHC and CESC though further studies are required to validate the prognostic value in larger, more diverse patient populations. The results also show the potential for GOT1/GOT2 in anti-cancer drug development. We identified the targets of several cancer-associated miRNAs as enriched in genes encoding GOT1 and GOT2 interacting proteins. Among them, we identified mir-34a-5p, a key miRNA in the regulation of P53 (126, 127) and an emerging cancer therapeutic target (152). While a first-in-human Phase I clinical trial of this miRNA was terminated prematurely due to serious adverse immune-mediated events (153), the recent development of a fully modified version of mir-34a with enhanced stability, activity and anti-tumor efficacy resulting in complete cures of some mice has ignited increased interest in therapeutic development (154).

## Research limitations and future directions

It is important to note that the work reported in this study is based on analyses of limited public transcriptional and proteomic datasets. The transcription levels of genes are not always directly correlated with the protein levels. While direct analysis of GOT1/GOT2 mRNA and protein levels from matched tumor samples would be ideal, large-scale mRNA-protein datasets across tumors are currently lacking. The use of bulk cell transcriptome data which does not differentiate expression in cancer cells vs other cells including immune cells near the tumor as well as the tumor microenvironment is another limitation. Furthermore, CIBERSORT has low sensitivity which likely fails to detect low-abundance immune cells such as Tregs and follicular helper T-cells influence prognosis. Verification with single cell RNAseq will be required in future. The associations between GOT1 and GOT2 with prognosis may be dependent on other properties of the tumors that were not considered in this study. For example, the tumor microenvironment, surrounding cell types, mutation or expression status of other genes and patient features including demographics, tumor grade, lymph node metastasis and treatment methods. In addition to being noisy, experimentally available protein-protein interaction datasets are often collected in a single cell type and some of the interactions have only been reported by a single study hence lack replication. It is possible that many experimentally determined interactions are absent in other cell types and maybe highly regulated developmentally, temporally and in different tumors. The analysis of infiltrating immune cells will in future require experimental validation for any associations with the expression of GOT1 and GOT2 as the current study utilized computational techniques. Future studies validating the results of this study in independent cohorts will also be important.

### Conclusion

In conclusion, this study indicates that both GOT1 and GOT2 are widely expression across several tissues and are perturbed at the transcriptional and proteomic levels in several tumors. In terms of potential clinical significance, the results show that GOT1 and GOT2 expression can serve as potential prognostic markers in specific cancers including LIHC and CESC. Finally, the findings suggest mir34a-GOT1 axis as a potential target for tumor metabolic therapy.

## Data Availability

Publicly available datasets were analyzed in this study. This data can be found here: The Genotype-Tissue Expression (GTEx) Project was supported by the Common Fund of the Office of the Director of the National Institutes of Health, and by NCI, NHGRI, NHLBI, NIDA, NIMH, and NINDS. The data used for the analyses described in this article were obtained from: the GTEx Portal on 05/11/23. The pan-cancer analyses presented in this work were in part based upon data generated by the TCGA Research Network: https://www.cancer.gov/tcga.
