# Supplementary material for: Pan-cancer molecular signatures connecting aspartate transaminase to cancer prognosis, metabolic and immune signatures
Source: Front Oncol. 2026 May 20;16:1727389. doi: 10.3389/fonc.2026.1727389 (PMC13229698; doi:10.3389/fonc.2026.1727389)
Supplement: Supplementary file 1 [file DataSheet1.docx]

**Supplementary Information**

**Supplementary Methods**

**Data Table for Normal vs. matched Tumor samples from TCGA (Fig. 1)**

| **Cancer Type** | **Normal samples** | **Tumor samples** |
| --- | --- | --- |
| BLCA | 19 | 408 |
| BRCA | 114 | 1097 |
| CESC | 3 | 305 |
| CHOL | 9 | 36 |
| COAD | 41 | 286 |
| ESCA | 11 | 184 |
| GBM | 0 | 256 |
| HNSC | 44 | 520 |
| KICH | 25 | 67 |
| KIRC | 72 | 533 |
| KIRP | 32 | 290 |
| LIHC | 50 | 371 |
| LUAD | 59 | 515 |
| LUSC | 52 | 503 |
| PAAD | 4 | 178 |
| PRAD | 52 | 697 |
| PCPG | 3 | 179 |
| READ | 10 | 166 |
| SARC | 2 | 260 |
| SKCM | 1 | 472 |
| THCA | 59 | 505 |
| THYM | 2 | 120 |
| STAD | 34 | 415 |
| UCEC | 35 | 456 |

**Supplementary Figure 1**

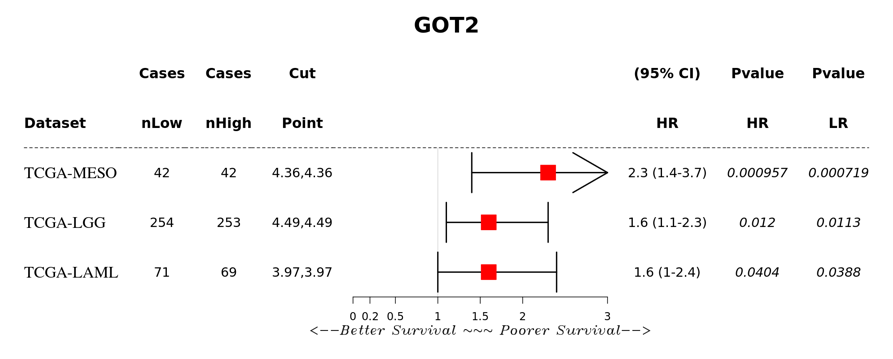


**Supplementary Fig. 2**

**Supplementary Figure 3**

P = 3.9e-06

P = 0.001

P = 0.2

P = 0.0003

P = 0.02

P = 0.7

P = 0.9

P = 0.8

P = 0.1

P = 0.7

P = 0.01

P = 0.0002

P = 0.02

P = 0.9

P = 0.5

P = 0.002

P = 0.96

P = 4.7e-06

P = 0.4

P = 0.42

P = 0.0004

P = 0.6

P = 0.3

P = 0.5

P = 0.003

P = 0.2

P = 0.3

P = 0.1

P = 0.02

P = 0.4

P = 0.4

P = 0.05

**Supplementary Table 1:** Protein expression of GOT1 across pan-cancer subtype 2 (“S” subtypes)

| **Comparison** | **Statistical significance** |
| --- | --- |
| **S1-vs-S2** | **1.05896551482327E-06** |
| **S1-vs-S3** | **1.242520E-04** |
| S1-vs-S4 | 5.505592E-01 |
| **S1-vs-S5** | **5.9719789101792E-06** |
| **S1-vs-S6** | **4.646380E-03** |
| **S1-vs-S7** | **3.12188814823019E-08** |
| **S1-vs-S8** | **2.276876E-04** |
| **S1-vs-S9** | **3.96877412611994E-06** |
| **S1-vs-S10** | **4.741356E-04** |
| **S1-vs-S11** | **1.633536E-03** |
| S2-vs-S3 | 2.369850E-01 |
| **S2-vs-S4** | **3.17981524479123E-16** |
| S2-vs-S5 | 5.156640E-01 |
| **S2-vs-S6** | **6.612786E-04** |
| S2-vs-S7 | 7.886367E-02 |
| **S2-vs-S8** | **3.014693E-02** |
| S2-vs-S9 | 9.601194E-01 |
| **S2-vs-S10** | **1.100498E-03** |
| **S2-vs-S11** | **1.29784997001995E-19** |
| **S3-vs-S4** | **1.70534834722379E-06** |
| S3-vs-S5 | 5.170667E-01 |
| S3-vs-S6 | 1.034156E-01 |
| **S3-vs-S7** | **1.648686E-02** |
| S3-vs-S8 | 5.707774E-01 |
| S3-vs-S9 | 3.008542E-01 |
| S3-vs-S10 | 2.604719E-01 |
| **S3-vs-S11** | **6.73739509105303E-14** |
| **S4-vs-S5** | **7.50751698614308E-12** |
| **S4-vs-S6** | **4.085093E-04** |
| **S4-vs-S7** | **1.11359845842929E-19** |
| **S4-vs-S8** | **3.38688743156801E-07** |
| **S4-vs-S9** | **1.09681640140354E-09** |
| **S4-vs-S10** | **1.27986611626931E-07** |
| **S4-vs-S11** | **1.39115723141316E-06** |
| **S5-vs-S6** | **7.523038E-03** |
| **S5-vs-S7** | **2.696215E-02** |
| S5-vs-S8 | 1.450617E-01 |
| S5-vs-S9 | 5.849375E-01 |
| **S5-vs-S10** | **2.045250E-02** |
| **S5-vs-S11** | **7.3084011011793E-18** |
| **S6-vs-S7** | **3.96589539289335E-06** |
| S6-vs-S8 | 2.118035E-01 |
| **S6-vs-S9** | **5.208718E-03** |
| S6-vs-S10 | 4.144923E-01 |
| **S6-vs-S11** | **1.13815282957725E-11** |
| **S7-vs-S8** | **4.173384E-04** |
| S7-vs-S9 | 1.960367E-01 |
| **S7-vs-S10** | **2.59025287743712E-06** |
| **S7-vs-S11** | **3.24352826697589E-22** |
| S8-vs-S9 | 7.912348E-02 |
| S8-vs-S10 | 5.392389E-01 |
| **S8-vs-S11** | **1.24391885617478E-14** |
| **S9-vs-S10** | **1.442506E-02** |
| **S9-vs-S11** | **6.11988917222274E-17** |
| **S10-vs-S11** | **1.37401493343956E-14** |

**Supplementary Table 2:** Protein expression of GOT1 across pan-cancer “K” subtypes

| **Comparison** | **Statistical significance** |
| --- | --- |
| **K1-vs-K2** | **2.049439E-04** |
| **K1-vs-K3** | **2.904263E-03** |
| **K1-vs-K4** | **4.569760E-02** |
| K1-vs-K5 | 4.507418E-01 |
| **K1-vs-K6** | **2.591916E-04** |
| **K1-vs-K7** | **7.214333E-03** |
| K1-vs-K8 | 1.730756E-01 |
| K1-vs-K9 | 1.705807E-01 |
| **K1-vs-K10** | **3.392581E-02** |
| K2-vs-K3 | 2.357407E-01 |
| K2-vs-K4 | 5.160282E-02 |
| **K2-vs-K5** | **7.64357514696217E-06** |
| K2-vs-K6 | 5.399513E-01 |
| **K2-vs-K7** | **3.553567E-02** |
| **K2-vs-K8** | **1.852669E-03** |
| **K2-vs-K9** | **1.399825E-04** |
| **K2-vs-K10** | **4.617745E-02** |
| K3-vs-K4 | 3.355686E-01 |
| **K3-vs-K5** | **4.622581E-04** |
| K3-vs-K6 | 3.999099E-01 |
| K3-vs-K7 | 4.262346E-01 |
| **K3-vs-K8** | **3.608940E-02** |
| **K3-vs-K9** | **6.304371E-03** |
| K3-vs-K10 | 3.408621E-01 |
| K4-vs-K5 | 6.364198E-02 |
| K4-vs-K6 | 7.893589E-02 |
| K4-vs-K7 | 6.719550E-01 |
| K4-vs-K8 | 3.704872E-01 |
| K4-vs-K9 | 2.313805E-01 |
| K4-vs-K10 | 9.543790E-01 |
| **K5-vs-K6** | **2.05664495503882E-08** |
| **K5-vs-K7** | **4.729602E-04** |
| K5-vs-K8 | 3.155248E-01 |
| K5-vs-K9 | 2.820547E-01 |
| **K5-vs-K10** | **3.846192E-02** |
| **K6-vs-K7** | **3.335331E-02** |
| **K6-vs-K8** | **1.321725E-03** |
| **K6-vs-K9** | **6.47822039763312E-06** |
| K6-vs-K10 | 6.778317E-02 |
| K7-vs-K8 | 9.389489E-02 |
| **K7-vs-K9** | **1.303132E-02** |
| K7-vs-K10 | 7.038927E-01 |
| K8-vs-K9 | 8.399931E-01 |
| K8-vs-K10 | 3.145936E-01 |
| K9-vs-K10 | 1.742075E-01 |

**Supplementary Table 3:** Protein expression of GOT2 across pan-cancer subtype 2 (“S” subtypes)

| **Comparison** | **Statistical significance** |
| --- | --- |
| S1-vs-S2 | 2.764144E-01 |
| S1-vs-S3 | 5.428634E-01 |
| **S1-vs-S4** | **2.41212357291803E-08** |
| **S1-vs-S5** | **1.148701E-02** |
| S1-vs-S6 | 1.112234E-01 |
| S1-vs-S7 | 3.247455E-01 |
| **S1-vs-S8** | **2.582699E-02** |
| S1-vs-S9 | 2.769208E-01 |
| S1-vs-S10 | 1.805810E-01 |
| **S1-vs-S11** | **1.11476156919406E-07** |
| S2-vs-S3 | 5.654427E-01 |
| **S2-vs-S4** | **8.49541439613905E-19** |
| **S2-vs-S5** | **7.94529466203424E-08** |
| S2-vs-S6 | 3.946192E-01 |
| **S2-vs-S7** | **1.322986E-03** |
| S2-vs-S8 | 5.268595E-02 |
| S2-vs-S9 | 8.911707E-01 |
| S2-vs-S10 | 6.999764E-01 |
| **S2-vs-S11** | **1.85594834366474E-09** |
| **S3-vs-S4** | **3.14018600300725E-13** |
| **S3-vs-S5** | **4.92663722266389E-05** |
| S3-vs-S6 | 2.074913E-01 |
| **S3-vs-S7** | **3.329282E-02** |
| **S3-vs-S8** | **3.220853E-02** |
| S3-vs-S9 | 5.418423E-01 |
| S3-vs-S10 | 3.613515E-01 |
| **S3-vs-S11** | **4.06302656131284E-09** |
| **S4-vs-S5** | **9.73843895147848E-32** |
| **S4-vs-S6** | **7.28745103435624E-15** |
| **S4-vs-S7** | **1.77358363987216E-28** |
| **S4-vs-S8** | **4.00507519266657E-12** |
| **S4-vs-S9** | **3.32964069691466E-11** |
| **S4-vs-S10** | **9.25502650607317E-22** |
| S4-vs-S11 | 6.060741E-01 |
| **S5-vs-S6** | **2.9024869959487E-09** |
| **S5-vs-S7** | **1.928343E-02** |
| **S5-vs-S8** | **7.79326443356387E-12** |
| **S5-vs-S9** | **4.0850753367784E-06** |
| **S5-vs-S10** | **1.83276691644722E-09** |
| **S5-vs-S11** | **1.18975466458995E-18** |
| **S6-vs-S7** | **9.54038773671492E-05** |
| S6-vs-S8 | 3.000943E-01 |
| S6-vs-S9 | 5.784134E-01 |
| S6-vs-S10 | 5.754463E-01 |
| **S6-vs-S11** | **4.88737742534135E-08** |
| **S7-vs-S8** | **8.02086442103514E-07** |
| **S7-vs-S9** | **5.878561E-03** |
| **S7-vs-S10** | **1.294477E-04** |
| **S7-vs-S11** | **1.54654873609547E-14** |
| S8-vs-S9 | 1.556448E-01 |
| S8-vs-S10 | 8.031637E-02 |
| **S8-vs-S11** | **1.09129085965805E-06** |
| S9-vs-S10 | 8.787951E-01 |
| **S9-vs-S11** | **7.23896018928741E-08** |
| **S10-vs-S11** | **2.25109659096499E-09** |

**Supplementary Table 4:** Protein expression of GOT2 across pan-cancer “K” subtypes

| **Comparison** | **Statistical significance** |
| --- | --- |
| **K1-vs-K2** | **4.882929E-02** |
| K1-vs-K3 | 6.583243E-01 |
| K1-vs-K4 | 2.306996E-01 |
| **K1-vs-K5** | **4.082383E-03** |
| K1-vs-K6 | 2.452687E-01 |
| K1-vs-K7 | 6.139935E-01 |
| K1-vs-K8 | 1.967151E-01 |
| K1-vs-K9 | 2.040442E-01 |
| **K1-vs-K10** | **7.864264E-04** |
| **K2-vs-K3** | **1.074099E-03** |
| K2-vs-K4 | 4.259403E-01 |
| K2-vs-K5 | 2.133390E-01 |
| K2-vs-K6 | 2.094241E-01 |
| **K2-vs-K7** | **1.597597E-02** |
| K2-vs-K8 | 2.997606E-01 |
| K2-vs-K9 | 2.307810E-01 |
| **K2-vs-K10** | **3.992451E-02** |
| **K3-vs-K4** | **4.280462E-02** |
| **K3-vs-K5** | **8.08479731226368E-07** |
| **K3-vs-K6** | **2.073307E-02** |
| K3-vs-K7 | 1.288011E-01 |
| **K3-vs-K8** | **1.413581E-02** |
| **K3-vs-K9** | **1.102529E-02** |
| **K3-vs-K10** | **7.64441023061932E-07** |
| K4-vs-K5 | 5.893692E-02 |
| K4-vs-K6 | 8.172209E-01 |
| K4-vs-K7 | 2.675084E-01 |
| K4-vs-K8 | 9.517425E-01 |
| K4-vs-K9 | 8.873739E-01 |
| **K4-vs-K10** | **1.124012E-02** |
| **K5-vs-K6** | **5.499208E-03** |
| **K5-vs-K7** | **1.01760338526256E-05** |
| **K5-vs-K8** | **1.303625E-02** |
| **K5-vs-K9** | **5.177650E-03** |
| K5-vs-K10 | 2.579052E-01 |
| K6-vs-K7 | 2.374458E-01 |
| K6-vs-K8 | 8.315453E-01 |
| K6-vs-K9 | 8.981846E-01 |
| **K6-vs-K10** | **1.040275E-03** |
| K7-vs-K8 | 1.671853E-01 |
| K7-vs-K9 | 1.562240E-01 |
| **K7-vs-K10** | **1.31289610484227E-05** |
| K8-vs-K9 | 9.204135E-01 |
| **K8-vs-K10** | **2.240220E-03** |
| **K9-vs-K10** | **1.031038E-03** |

**Supplementary Table 5:** GOT1 protein-protein interactions from BioGrid

**Supplementary Table 6:** GOT2 protein-protein interactions from BioGrid

**Supplementary Table 7:** Top 10 significant p-values and q-values for GOT1 PPis based on DisGeNET

| **Table of top 10 significant p-values and q-values for GOT1 PPis based on DisGeNET** | | | |
| --- | --- | --- | --- |
| **term** | **p-value** | **q-value** | **overlap_genes** |
| Dermatologic disorders | 5.26E-16 | 2.38E-12 | [FLG, IL1RN, FH, CALML5, ALOX12B, PLG, DMKN, EGFR, TGM1, S100A7A, NME1-NME2, POF1B, LMNA, HMOX1, IL36RN, NANS, TGM5, TGM3, DSP, CDSN, DDX58, NME2, NME1, DSG1, DSG3, CD44, IVL, DSC2, S100A8, BLVRA, S100A7, PLEC] |
| Neoplasm Metastasis | 8.76E-14 | 1.99E-10 | [IL1RN, STEAP4, SDR9C7, ENO2, ICAM1, PYCARD, LGALS3, CDH1, TRIM29, TAP1, ALDH3A1, MTAP, CLIP1, AGTR1, CMPK1, PKP1, HPRT1, PKP3, EZR, S100A9, DSC2, S100A8, S100A7, MACF1, CFH, IQGAP1, NDRG1, NME1-NME2, CYB5R3, HMOX1, SFN, S100A14, TRAP1, CDSN, IDH1, ARHGAP24, ESR2, TF, HAL, AKR1B10, BCL6, ALB, GRB2, RAB5A, BAP1, SERPINA3, CLIC3, ARHGAP1, TNFAIP2, S100A7A, ANXA6, QSOX1, PHGDH, TMEM189-UBE2V1, ACP1, CCR2, DSP, SERPINB4, CBR1, ANXA1, TPI1, SERPINB2, DBNL, ELOVL4, DDX58, NME2, APOA1, TYK2, F2, SERPINB5, NME1, ALDH1A3, PKM, KIF2C, LCP1, DSG3, ACPP, CD44, PLEC, LTF, FH, MVP, CAPG, PLG, TYMS, SBSN, CRIP1, EGFR, TYMP, NXF1, RAB25, ABI3BP, LMNA, XDH, NQO1, LGALS7B, GSN, TXNRD2, AKR1C2, HSPE1, ...] |
| Squamous cell carcinoma | 9.35E-13 | 1.41E-09 | [IL1RN, DMKN, ICAM1, PYCARD, TGM1, S100A7A, LGALS3, CDH1, TRIM29, ANXA8, NANS, TGM3, CCR2, SERPINB3, DSP, SERPINB4, ANXA1, TPI1, SERPINB2, NME2, TALDO1, SERPINB5, NME1, MTAP, PKM, PKP1, DSG1, DSG3, EZR, MAPRE2, S100A9, CD44, IVL, DSC2, S100A8, S100A7, FLG, CRABP2, ALOX12B, PLG, TYMS, PPL, NDRG1, EGFR, TYMP, SNX3, NME1-NME2, HMOX1, SFN, S100A14, NQO1, LGALS7B, GSN, JUP, IDH1, TREX2, GSR, AKR1C2, KLK10, ARHGAP24, ESR2, SOD1, HAL, AKR1B10] |
| Adenocarcinoma of lung (disorder) | 3.52E-12 | 4.00E-09 | [CRABP2, CFH, ARHGAP1, CAPG, ENO2, TYMS, NDRG1, EGFR, ICAM1, S100A7A, CDH1, LMNA, UBQLN1, HMOX1, SFN, S100A14, TGM5, XDH, SERPINB3, SERPINB4, NQO1, CBR1, ANXA1, TPI1, GSN, IDH1, APOA1, ARHGAP24, ESR2, NME1, SOD1, ALDH3A1, MTAP, TF, HAL, AKR1B10, PKM, ALB, CD9, BLVRB, GRB2, HPRT1, PKP3, EZR, S100A9, RAB5A, BAP1, CD44] |
| Contact hypersensitivity | 1.02E-11 | 8.87E-09 | [SERPINB3, FLG, SERPINB4, NQO1, GSR, AKR1C2, SOD1, AKR1B10, PIR, HMOX1, KIF2C, CD44, S100A8] |
| Psoriasis | 1.59E-11 | 8.87E-09 | [FLG, IL1RN, CALML5, PLG, CTSV, EGFR, ICAM1, C3, TGM1, S100A7A, NME1-NME2, C4A, NXF1, ANXA6, HMOX1, IL36RN, S100A14, TGM5, TGM3, CCR2, SERPINB3, CAST, SERPINB4, SERPINB1, CDSN, DDX58, NME2, IL36G, TAP1, APOA1, TYK2, SERPINB8, NME1, DSG1, S100A9, IVL, S100A8, S100A7] |
| Squamous cell carcinoma of esophagus | 1.63E-11 | 8.87E-09 | [IL1RN, CRABP2, TNFAIP2, SDR9C7, PLG, IQGAP1, TYMS, SBSN, PPL, EVPL, NDRG1, EGFR, TYMP, RAB25, CDH1, HMOX1, SFN, S100A14, NEFH, TGM3, SERPINB3, DSP, TRAP1, NQO1, ANXA1, TPI1, SERPINB2, AKR1C2, SERPINB5, ARHGAP24, ESR2, NME1, MTAP, PKM, PSME2, BLVRB, GRB2, EZR, ALDH7A1, BAP1, CD44, DSC2, S100A8] |
| Adenocarcinoma | 1.92E-11 | 8.87E-09 | [IL1RN, ICAM1, PYCARD, LGALS3, CDH1, PHGDH, ANXA8, ACP1, SERPINB3, DSP, SERPINB4, ANXA1, TPI1, SERPINB2, NME2, APOA1, SERPINB5, NME1, ALDH1A3, MTAP, PKM, AGTR1, PSME2, DSG3, ACPP, S100A9, CD44, DSC2, S100A8, PLEC, CRABP2, CFH, CAPG, PLG, IQGAP1, TYMS, NDRG1, EGFR, TYMP, NME1-NME2, IGKC, RAB25, LMNA, HMOX1, SFN, NQO1, GSN, IDH1, GSR, AKR1C2, KLK10, ARHGAP24, ESR2, SOD1, AKR1B10, ALB, CD9, S100P] |
| Acanthosis | 1.93E-11 | 8.87E-09 | [TGM1, CAST, IL1RN, JUP, DSG1, ALOX12B, IL36RN, SERPINB7, SERPINB8, EGFR] |
| Primary malignant neoplasm of lung | 1.96E-11 | 8.87E-09 | [IL1RN, ENO2, ICAM1, PYCARD, TGM1, S100A7A, LGALS3, CDH1, ACP1, CCR2, SERPINB3, DSP, SERPINB4, CBR1, ANXA1, SERPINB2, NME2, TAP1, PTGR1, SERPINB5, NME1, ALDH1A3, MTAP, PKM, AGTR1, KIF2C, HPRT1, PKP3, DSG3, EZR, ALDH7A1, CD44, S100A8, LTF, TSTA3, CRABP2, CFH, SHMT2, MVP, ALOX12B, PLG, TYMS, SBSN, NDRG1, EGFR, TYMP, NXF1, RAB25, ABI3BP, LMNA, HMOX1, SFN, S100A14, XDH, NQO1, RBM39, GSN, ZNF185, TXNRD2, ARHGAP24, ESR2, SOD1, CUL4A, AKR1B10, ALB, CD9, GRB2, RAB5A, BAP1] |

**Supplementary Table 8:** Top 10 significant p-values and q-values for GOT2 PPis based on DisGeNET

| **Table of top 10 significant p-values and q-values for GOT2 PPis based on DisGeNET** | | | |
| --- | --- | --- | --- |
| **term** | **p-value** | **q-value** | **overlap_genes** |
| Liver carcinoma | 2.29E-15 | 6.34E-12 | [GPI, SERPINA3, MPG, PEBP1, ENO1, CTSS, LOXL2, AIFM1, MYC, CTSH, MAP3K7, SERPINB4, ACOT7, MAP2K2, ANXA1, TPI1, PARP1, CTNNBIP1, RIPK4, PTGR1, CDC34, PPIB, TKT, GAPDH, BIRC3, CD274, APCS, HDAC5, CUL7, VCP, PSMD14, TXN, ADH5, HDAC6, SEC14L2, PRDX5, AGR2, THEM4, APOD, E2F4, NTRK1, HSPA8, NQO1, RBM39, RRM1, CCL21, FUS, FAH, COPS5, CDK2, PIN1, CALR] |
| Primary malignant neoplasm of lung | 1.54E-10 | 2.13E-07 | [CD274, APCS, MPG, TXN, ENO1, PRKCZ, ADH5, LOXL2, MYLK, SEC14L2, AIFM1, MB, MYC, CFL1, AGR2, THEM4, E2F4, MAP3K7, NTRK1, HSPA8, SERPINB4, NQO1, RBM39, RRM1, MAP2K2, ANXA1, CCL21, PARP1, FUS, PTGR1, CDC34, CDK2, PIN1, GAPDH, BIRC3] |
| Malignant neoplasm of lung | 2.93E-10 | 2.71E-07 | [CD274, APCS, MPG, COX17, TXN, ENO1, PRKCZ, ADH5, LOXL2, MYLK, SEC14L2, AIFM1, MB, MYC, CFL1, AGR2, THEM4, E2F4, MAP3K7, NTRK1, HSPA8, SERPINB4, NQO1, RBM39, RRM1, MAP2K2, ANXA1, CCL21, PARP1, FUS, PTGR1, EWSR1, CDK2, PIN1, GAPDH, BIRC3] |
| Carcinogenesis | 5.17E-10 | 3.08E-07 | [CD274, GPI, HDAC5, VCP, PSMD14, MPG, COX17, PEBP1, TXN, ENO1, PRKCZ, ADH5, CTSS, HDAC6, SEC14L2, FGD5, PSMD4, MB, MYC, AGR2, THEM4, SHOC2, E2F4, BRD3, NTRK1, SERPINB4, NQO1, RBM39, RRM1, MAP2K2, ANXA1, CCL21, PARP1, FUS, MDH2, RIPK4, FAH, COPS5, BCL6, EWSR1, CDK2, PIN1, PRKD2, CALR, PPIB, GAPDH, BIRC3] |
| Neoplasm Metastasis | 5.56E-10 | 3.08E-07 | [CD274, GPI, SERPINA3, APCS, VCP, PEBP1, SDR9C7, TXN, ENO1, CTSS, HDAC6, LOXL2, MYLK, SEC14L2, PSMD4, AIFM1, MB, MYC, CFL1, AGR2, CTSH, SHOC2, E2F4, MAP3K7, NTRK1, HSPA8, SERPINB4, NQO1, RRM1, MAP2K2, ANXA1, TPI1, CCL21, PARP1, FUS, CTNNBIP1, COPS5, BCL6, EWSR1, CDK2, NBR1, PIN1, CALR, TKT, GAPDH, BIRC3] |
| Neuroblastoma | 1.01E-09 | 4.64E-07 | [CD274, HDAC5, CUL7, PEBP1, GLRX, TXN, ENO1, PRKCZ, HDAC6, PRDX5, PSMD4, MB, MYC, CFL1, APOD, NTRK1, HSPA8, NQO1, TPI1, CCL21, PARP1, FUS, CTNNBIP1, EWSR1, CDK2, PIN1, CALR, TARDBP, GAPDH] |
| Carcinoma of lung | 1.68E-09 | 6.64E-07 | [CD274, APCS, MPG, TXN, ENO1, PRKCZ, ADH5, LOXL2, MYLK, SEC14L2, AIFM1, MB, MYC, CFL1, AGR2, THEM4, E2F4, MAP3K7, NTRK1, HSPA8, SERPINB4, NQO1, RBM39, RRM1, MAP2K2, ANXA1, CCL21, PARP1, FUS, PTGR1, CDC34, CDK2, PIN1, GAPDH, BIRC3] |
| Non-Small Cell Lung Carcinoma | 2.20E-09 | 6.77E-07 | [CD274, HDAC5, CUL7, VCP, MPG, COX17, PEBP1, TXN, ENO1, CTSS, HDAC6, MYLK, SEC14L2, PRDX5, PSMD4, MB, MYC, CFL1, AGR2, TRIM67, NTRK1, SERPINB4, NQO1, RBM39, RRM1, MAP2K2, CCL21, PARP1, FUS, CDK2, PIN1, GAPDH, BIRC3] |
| Malignant neoplasm of breast | 2.20E-09 | 6.77E-07 | [GPI, SERPINA3, MPG, PEBP1, ENO1, LOXL2, MYLK, PSMD4, AIFM1, MB, MYC, CFL1, MAP3K7, BRD3, SERPINB4, ANXA1, TPI1, PARP1, PPIB, ZMYND11, GAPDH, BIRC3, CD274, APCS, VCP, PSMD14, TXN, PRKCZ, ADH5, HDAC6, SEC14L2, FGD5, PRDX5, AGR2, THEM4, APOD, E2F4, NTRK1, HSPA8, NQO1, RBM39, RRM1, CCL21, FUS, FAH, COPS5, BCL6, CDK2, NBR1, PIN1, CALR, TARDBP] |
| Central neuroblastoma | 2.65E-09 | 7.32E-07 | [CD274, HDAC5, CUL7, PEBP1, GLRX, TXN, ENO1, HDAC6, PRDX5, PSMD4, MB, MYC, CFL1, APOD, NTRK1, HSPA8, NQO1, TPI1, CCL21, PARP1, FUS, CTNNBIP1, EWSR1, CDK2, PIN1, CALR, TARDBP, GAPDH] |

**Supplementary Table 9: Top 10 significant p-values and q-values for GOT1 Transcription Factor PPIs**

| **term** | **p-value** | **q-value** | **overlap_genes** |
| --- | --- | --- | --- |
| ESR1 | 1.108185e-10 | 1.518213e-08 | [WDR1, SHMT2, MVP, ARHGAP1, IQGAP1, RPL6, EGFR, POF1B, LMNA, PGM2, SFN, PHGDH, ACAA1, HIST1H1B, DSP, TRAP1, SERPINB4, LGALS7B, RBM39, GSN, JUP, H2AFY, IDH1, NME2, TAP1, HSPE1, NCCRP1, ESR2, NME1, TF, DSG1, GRB2, EPPK1, EZR, S100A8, EZH2, S100A7, PLEC] |
| ATF2 | 3.766309e-06 | 2.579922e-04 | [FH, ACADVL, ANXA1, TPI1, OAT, H2AFY, SHMT2, SQRDL, HSPE1, PKM, RAB14, SFN, CD44, EZH2] |
| USF2 | 1.712457e-05 | 7.820222e-04 | [SERPINB3, SERPINB4, IGHG2, S100A9, S100A8] |
| ILF3 | 4.741048e-05 | 1.623809e-03 | [RBM39, JUP, GAA, NDRG1, RPL6, CHCHD4, NXF1, MOV10, PC, IGKC, RAB14, NUP35, GRB2, RAE1] |
| CBX3 | 1.438349e-04 | 3.941077e-03 | [WDR1, H2AFY, OXCT1, IDH1, LMNA, TALDO1, HSPE1, BAP1] |
| CTNNB1 | 9.442288e-04 | 2.155989e-02 | [MACF1, RBM39, JUP, IQGAP1, NDRG1, EGFR, ESR2, CUL4A, LGALS3, CDH1, LMNA, EZR, ACP1, EZH2] |
| RAD21 | 1.329435e-03 | 2.601894e-02 | [C3, CUL4A, TRAP1, CBR1, SERPINB1, CALML5, CALML3, S100A9, RPL6, HIST1H1B] |
| KRT7 | 1.925162e-03 | 3.296840e-02 | [IGHG1, GRB2, EGFR] |
| NR3C1 | 1.495964e-02 | 2.191502e-01 | [FGB, IL1RN, CLIP1, MVP, ALB, SFN, ACP1, TJP2] |
| PPARD | 1.599637e-02 | 2.191502e-01 | [JUP, BCL6, SHMT2] |

**Supplementary Table 10: Top 10 significant p-values and q-values for GOT2 Transcription Factor PPIs**

| **term** | **p-value** | **q-value** | **overlap_genes** |
| --- | --- | --- | --- |
| ESR1 | 5.684151e-13 | 1.051568e-10 | [HDAC5, VCP, MPG, ABAT, ENO1, PRKCZ, AIFM1, U2AF2, MYC, CFL1, SHOC2, APOD, HADH, HSPA8, SERPINB4, RBM39, PARP1, FUS, MDH2, COPS5, EWSR1, CDK2, TKT, GAPDH, EZH2] |
| TP53 | 1.625568e-09 | 1.503650e-07 | [NTRK1, HSPA8, NQO1, HDAC5, CUL7, VCP, PARP1, COX17, TXN, ADH5, COPS5, PSMD4, EWSR1, MYC, CDK2, PIN1, ZMYND11, GAPDH] |
| ATF2 | 2.586069e-08 | 1.594743e-06 | [PRDX5, ANXA1, TPI1, MDH2, CFL1, CDK2, ENO1, CALR, PPIB, GAPDH, EZH2] |
| HIF1A | 3.067008e-06 | 1.218770e-04 | [HDAC5, VCP, COPS5, CDC34, PARP1, MYC, HDAC6] |
| EP300 | 3.822734e-06 | 1.218770e-04 | [BCL6, PARP1, MPG, EWSR1, TNIP2, MYC, CDK2, PIN1, ENO1, GAPDH, PARK2, HDAC6] |
| CTNNB1 | 4.434997e-06 | 1.218770e-04 | [HSPA8, RBM39, COPS5, PARP1, FUS, MYC, CTNNBIP1, CDK2, PIN1, HDAC6, EZH2] |
| HCFC1 | 4.611563e-06 | 1.218770e-04 | [HDAC5, HSPA8, USP53, CDK2, E2F4, MAP3K7] |
| TRIM28 | 7.901592e-06 | 1.827243e-04 | [HDAC5, COPS5, CDC34, PARP1, MYC, MEPCE, CDK2, E2F4] |
| TARDBP | 1.087200e-05 | 2.234799e-04 | [VCP, U2AF2, FUS, PARK2, HDAC6] |
| POLR2A | 2.484885e-05 | 4.597037e-04 | [PARP1, EWSR1, FUS, U2AF2, MYC, CDK2, PIN1, EZH2] |

**Supplementary Table 11:** Top 10 significant p-values and q-values for GOT1 enriched miRNAs miRTarBase 2017

| **Term** | **p-value** | **q-value** | **Overlap_genes** |
| --- | --- | --- | --- |
| **hsa-miR-34a-5p** | **1.36E-08** | **0.000028** | [FH, MCFD2, CRABP2, ARHGAP1, CAPG, TYMS, NDRG1, SNX3, CASP14, SYNGR2, SFN, HIST1H2AG, EPS8L2, RAE1, HIST1H1B, DSP, SERPINB1, YOD1, MOV10, MTAP, DDAH2, AGTR1, CMPK1, PLIN3, GRB2, KIF2C, S100P, HPRT1, SHANK3, CD44, TJP2] |
| **hsa-miR-124-3p** | **4.70E-07** | **0.000487** | [FLG, ACADVL, CLIC3, GDA, SHMT2, MVP, ARHGAP1, IQGAP1, FAM129B, PYCARD, TGM1, FAM83H, CHRAC1, SYNGR2, TRIM29, LMNA, ANXA6, HMOX1, RBBP9, PGM2, QSOX1, ANXA8, YKT6, CAST, SERPINB2, DBNL, GSN, JUP, TREX2, AHNAK2, ANXA11, TYK2, MOV10, NECAP2, BCL6, GAN, AGTR1, PLIN3, PKP1, GRB2, KIF2C, TJP2, PLEC] |
| hsa-miR-1-3p | 9.78E-05 | 0.067616 | [ACADVL, ECHS1, OAT, ATL3, SRXN1, CAPG, EGFR, CHRAC1, OXCT1, HMOX1, PGM2, SFN, OSTF1, HIST1H1B, CAST, RBM39, ZNF185, JUP, AHNAK2, F2, PGD, SERPINB5, MOV10, PIR, KIF2C, LCP1, CD44] |
| hsa-miR-3659 | 2.19E-04 | 0.113857 | [PKM, TXNL1, VPS35, PIP4K2C, SHANK3, PLEC] |
| hsa-miR-4792 | 3.95E-04 | 0.15377 | [NME1-NME2, CRABP2, GAN, GSR, NME2, CPA4, PLEC] |
| hsa-miR-1304-5p | 4.45E-04 | 0.15377 | [PYCARD, CHCHD4, FAM83H, ALDH1A3, GSN, HMGCS1, FGG, A2ML1] |
| hsa-miR-4443 | 5.92E-04 | 0.175463 | [RAB5B, DCXR, HMOX1, SDR9C7, TRIM67, PLEC, FAM129B] |
| hsa-miR-30a-5p | 8.04E-04 | 0.184444 | [CAST, UBE2H, ANXA1, TPI1, FAHD1, JUP, IDH1, TNFAIP2, SERPINB5, EGFR, ESR2, SNX3, NT5C3A, UFM1, PKM, RAB14, WDR82, CDH1, PLIN3, LCP1, CD44] |
| hsa-miR-4430 | 8.89E-04 | 0.184444 | [RAB5B, TMEM40, F2, SNX3, PYCARD, NARS, MTAP, CCS, PKM, NAGK, GAN, HMOX1, PLIN3, QSOX1] |
| hsa-miR-3652 | 8.89E-04 | 0.184444 | [RAB5B, TMEM40, F2, SNX3, PYCARD, NARS, MTAP, CCS, PKM, NAGK, GAN, HMOX1, PLIN3, QSOX1] |

**Supplementary Table 12:** Top 10 significant p-values and q-values for GOT2 enriched miRNAs miRTarBase 2017

| **Term** | **p-value** | **q-value** | **Overlap_genes** |
| --- | --- | --- | --- |
| **hsa-miR-4476** | **0.000051** | **0.03613** | **[ALDH4A1, HDAC5, TPI1, CFL1, SDR9C7, CALR, TRIM67]** |
| **hsa-miR-6876-5p** | **0.000051** | **0.03613** | **[ALDH4A1, HDAC5, TPI1, CFL1, SDR9C7, CALR, TRIM67]** |
| hsa-miR-1301-3p | 0.000408 | 0.181755 | [VCP, PARP1, MDH2, CFL1, TXN, MYLK] |
| hsa-miR-4254 | 0.000511 | 0.181755 | [METTL14, CDC34, SDR9C7, PRKD2] |
| mmu-miR-127-3p | 0.001021 | 0.241095 | [GPI, BCL6] |
| mmu-miR-449a-5p | 0.001186 | 0.241095 | [MYC, PIN1, RAB11B] |
| hsa-miR-615-3p | 0.001244 | 0.241095 | [HSPA8, TPI1, CDC34, PARP1, EWSR1, FUS, MDH2, U2AF2, NBR1, E2F4, ENO1, GAPDH] |
| mmu-miR-34c-5p | 0.001633 | 0.241095 | [BCL6, PIN1, RAB11B] |
| hsa-miR-873-5p | 0.001847 | 0.241095 | [PRDX5, TPI1, CFL1, PIN1, ADH5, RAB11B] |
| hsa-miR-6859-5p | 0.001937 | 0.241095 | [VCP, CFL1, CDK2, PIN1, TRIM67] |
